# A structural review of foliar glands in *Passiflora* L. (Passifloraceae)

**DOI:** 10.1371/journal.pone.0187905

**Published:** 2017-11-14

**Authors:** Renata Cristina Cassimiro de Lemos, Delmira da Costa Silva, Gladys Flavia de Albuquerque Melo-de-Pinna

**Affiliations:** 1 Departamento de Botânica, Instituto de Biociências, Universidade de São Paulo, São Paulo, Brazil; 2 Departamento de Ciências Biológicas, Universidade Estadual de Santa Cruz, Bahia, Brazil; Indian Institute of Science, INDIA

## Abstract

Extrafloral glands in Passifloraceae species have aroused the interest of many researchers because of their wide morphological diversity. The present work analyzed the foliar glands on 34 species of *Passiflora* from samples containing glands in the petiole and foliar blade fixed in 50% solution of formaldehyde-ethanol-acetic acid and stored in a 70% ethanol solution. For anatomical analyses, part of the material was embedded in Paraplast, longitudinally sectioned and double stained with safranin and astra blue. Scanning electron microscopy analysis was also carried out. To analyze the presence of sugars in the secretion of foliar glands, a glucose strip test was used. Based on the results of morphological, anatomical and glucose strip tests, the foliar secretory glands in *Passiflora* can be grouped into two categories: Type I glands, defined as nectaries, can be elevated or flattened, and can have a sugar content high enough to be detected by the glucose strip test analysis. Type II glands are elevated and did not show a positive reaction to the glucose strip test. From an anatomical viewpoint, glands characterized as extrafloral nectaries show a multistratified secretory epidermis, typically followed by two flat layers of nectariferous parenchyma with dense content. Internal to these layers, vascular bundles are immersed in the subsecretory parenchyma and terminate in phloem cells. On the other hand, type II glands show a single layer of elongated secretory epidermal cells. Internal to this single layer, parenchyma and vascular tissue with both phloem and xylem elements can be observed. The analyzed species show a wide diversity of gland shape and distribution, and the combined analysis of morphology, anatomy and preliminary tests for the presence of glucose in the exudate in different *Passiflora* subgenera suggests the occurrence of two categories of glands: nectaries and resin glands.

## Introduction

Admired for the beautiful flowers and their edible fruits, the Passifloraceae Juss. Ex. Rouseel family is the object of study in different areas. *Passiflora* L. is the most representative genus in the Passifloraceae *s*.*s*. family, comprising more than 500 species [1–4]. According to the most recent infrageneric classification the family is subdivided in five subgenera, including *Astrophea* (DC.) Mast., *Deidamioides* (Harms) Killip, *Decaloba* (DC.) Rchb., *Passiflora* [1], and *Tetrapathea* (DC) P.S. Green, the latter restricted to Oceania [3].

The occurrence of extrafloral nectaries (EFN) in Passifloraceae species is very common, and their presence, as well as their shape, has been widely used as a diagnostic characteristic for species or species groups within the genus *Passiflora* [2–10]. EFN can be found at the petiole (petiolar nectaries) or in the foliar blade (laminar nectaries), occasionally being found in bracts and stipules [1, 2].

Nectaries are specialized structures that secrete a sugary substance known as nectar [11, 12], which is mainly composed of sucrose, fructose, amino acids, proteins and other trace components [12, 13]. In the present study, we use the term extrafloral nectary for the foliar nectaries found in Passifloraceae, following the nomenclature proposed by Caspary [14] who delimited these structures, whether floral or extrafloral, based solely on their distinct position.

The petiolar EFN are one of the main characteristics used to identify *Passiflora* species. They can appear as scars or protrusions, and they can also resemble small barrels, trumpets or spoons [2]. Laminar nectaries also occur in various forms, such as glandular marginal teeth, isolated marginal glands, sub-marginal glands, and as ocellus between the three major veins, or even dispersed throughout the abaxial surface of the foliar blade [2].

According to Solereder [15], the leaves of some *Passiflora* and *Adenia* Forssk. species can also have glandular spots at the abaxial surface, which have an external appearance of rounded brown spots. When observed in cross-section, the glandular spots show many layers of elongated epidermal cells at the glandular region, which are followed by tissue with several crystal formations. The author also suggests that the petiolar glands, which are frequently observed in *Passiflora*, are modifications of these glandular spots.

In an extensive work about the species of Passifloraceae in the American continent, Killip [5] described diverse shapes of glands. Glands described as globose, clavate, patelliform, sessile or stipitate, orbicular, and linguliform were mentioned as elevated nectaries by Zimmermann [16]. The ones reported by Killip [5] as scar-shaped could be described as embedded or flattened nectaries according to the classifications of Zimmermann [16], although in this case a more precise description would be needed. Durkee [17] characterized the structure of EFN of nine *Passiflora* species. In this study, the author points out that petiolar nectaries show a variety of sizes and morphologies, usually distinguishable by the organ in which they are found. Thus, they fit the classification of elevated nectaries proposed by Zimmermann [16]. On the other hand, nectaries of the foliar blade were described as embedded in the abaxial surface of the leaf, a type added to Zimmermann´s [16] classification by Elias [11].

Based on EFN studies already carried out in *Passiflora*, these nectaries could be described as vascularized, according to Elias [11]. From an anatomical viewpoint, the structure of the EFN in *Passiflora* is quite similar to that of species already reported. Therefore, considering that various authors have used different nomenclature, three different and specialized regions are usually recognized in these types of nectaries: secretory epidermis, where the nectar is liberated to the exterior and where stomata and trichomes may be either present or absent; secretory parenchyma that produces or stores the nectar, and is composed of layers of small cells with a dense content, located immediately under the epidermis; and subsecretory parenchyma, which has large and fewer juxtaposed cells [13, 18, 19].

Besides the presence of nectaries, the occurrence of a glandular type referred to as “gland-tipped hairs”, “sticky glands” and “sticky hairs” are also reported [2, 5, 8]. This glandular type seems to be restricted to some species of the *Dysosmia* DC. section [15], from subgenus *Passiflora*, that shows a characteristic absence of petiolar glands, being replaced with “gland-tipped hairs” [2, 8]. Studying the development of *Passiflora foetida* L., Roth [20] described this glandular type as an EFN. However, Durkee *et al*. [21] were not able to find sugars in the chemical composition of the exudate. The authors verified that the exudate was soluble in ethanol and xylene, but not in water, and they also found osmiophilic material within the vacuole of secretory cells. According to the authors, *Passiflora foetida* shows a variability in its gland morphology and physiology that can be regarded as a transition from a true EFN to lipophilic secretory glands, thus naming them as resin glands.

Detailed investigation to elucidate the morphological and anatomical structures of foliar glands in *Passiflora* has not been conducted. To better understand the diversity of secretory glands, the current study was aimed at comparative morphological and anatomical analyses associated with the presence of glucose in the exudate, as determined by the glucose strip test. We described two categories of secretory glands and discuss the use of terms regarding the different shapes.

## Materials and methods

Thirty-four species were selected from the subgenera *Astrophea* (DC.) Mast. (1 species), *Deidamioides* (Harms) Killip (2 species), *Decaloba* (DC.) Rchb. (5 species) and *Passiflora* (26 species) (Table 1), and special attention was given to the subgenera *Passiflora*, given its greater diversity. Part of the analyzed species was cultivated in a greenhouse at the Institute of Biosciences of the University of São Paulo. Another part was obtained from cultivation at Embrapa Cerrados (Brasilia, Federal District, Brazil), from a greenhouse cultivation at the State University of Santa Cruz (UESC, Ilhéus, Bahia, Brazil), and from the greenhouse of a private collector. One species was obtained from its natural environment (S1 Table). The voucher material was deposited at the Herbarium of the Botany Department at the University of São Paulo (SPF). Voucher numbers of private collectors were also used for obtaining information about where the species were originally sampled (S1 Table).

**Table 1:** Distribution, shapes, gland classification and anatomical features of foliar glands in the analysed species of *Passiflora* L.

Species	Gland distribution	Gland shapes	Gland classification	Secretory epidermis composition	Number of layers of secretory parenchyma
**Subgenus *Astrophea***					
*P*. *haematostigma* Mart. ex Mast.	Petiole	Ellipsoid	elevated nectaries	4–6 layers of elongated cells, and occurrence of trichomes	2 layers of cells
	Margins of leaf blade	Elliptic-lenticular	elevated nectaries	3–4 layers of elongated cells, and occurrence of trichomes	1–2 layers of cells
**Subgenus *Decaloba***					
*P*. *ferruginea* Mast.	Petiole	Crateriform	elevated nectaries	4–6 layers of short cells	8–10 layers of cells
	Abaxial surface of leaf blade	Concave ocellus to convex ocellus	flattened nectaries	6–10 layers of short cells	3–5 layers of cells
*P*. *misera* Kunth	Abaxial surface of leaf blade	Concave ocellus to convex ocellus	flattened nectaries	4–8 layers of short cells	2–4 layers of cells
*P*. *morifolia* Mast.	Petiole	Cotyliform	elevated nectaries	6–8 layers of short cells	4–6 layers of cells
*P*. *organensis* Gardner	Abaxial surface of leaf blade	Concave ocellus to convex ocellus	flattened nectaries	4–6 layers of short cells	2–4 layers of cells
*P*. *suberosa* L.	Petiole	Crateriform	elevated nectaries	6–8 layers of short cells	2–6 layers of cells
**Subgenus *Deidamioides***					
*P*. *contracta* Vitta	Petiole	Ellipsoid	elevated nectaries	4–6 layers of elongated cells	2–3 layers of cells
	Abaxial surface of leaf blade	Convex ocellus	flattened nectaries	3–4 layers of elongated cells	
*P*. *deidamioides* Harms	Petiole	Elliptic-patelliform	elevated nectaries	4 layers of short cells	2–3 layers of cells
	Petiolule	Patelliform	elevated nectaries	4–8 layers of short cells	
**Subgenus *Passiflora***					
*Passiflora actinia* Hook	Petiole	Obconical	elevated nectaries	4–6 layers of short to elongated cells	2–3 layers of cells
*P*. *ambigua* Hemsl	Petiole	Ellipsoid	elevated nectaries	1–2 layers of elongated cells	2–3 layers of cells
*P*. *arida* (Mast. & Rose) Killip	Petiole	Terete	resin glands	1 layer of elongated cells	-
	Dispersed on both surfaces of leaf blade				
*P*. *coccinea* Aubl.	Petiole	Spheroidal	elevated nectaries	2–3 layers of elongated cells, and occurrence of trichomes	1–2 layers of cells
	Abaxial surface of leaf blade	Lenticular	elevated nectaries		
*P*. *edmundoi* Sacco	Petiole	Obconical long-stipitate	elevated nectaries	1–3 layers of elongated cells	2–3 layers of cells
	Margins of leaf blade	Obconical short-stipitate	elevated nectaries	1–2 layers of elongated cells	
*P*. *eichleriana* Mast.	Petiole	Obconical short-stipitate	elevated nectaries	2 layers of elongated cells	2–3 layers of cells
	Margins of leaf blade	Elliptic-lenticular	elevated nectaries	2–4 layers of elongated cells	
*P*. *elegans* Mast.	Petiole	Obconical	elevated nectaries	4–8 layers of short to elongated cells	2–3 layers of cells
	Margins of leaf blade	Obconical	elevated nectaries	6–8 layers of short cells	
*P*. *foetida* L.	Petiole	Pyriforme long-stipitate	resin glands	1 layer of elongated cells	-
	Dispersed on both surfaces of leaf blade				
*P*. *galbana* Mast.	Petiole	Spheroidal	elevated nectaries	1–2 layers of elongated cells	2 layers of cells
	Margins of leaf blade	Concave ocellus	flattened nectaries		
*P*. *gardneri* Mast.	Margins of leaf blade	Lenticular	elevated nectaries	1–2 layers of elongated cells	2 layers of cells
*P*. *incarnata* L.	Petiole	Elliptic-patelliform	elevated nectaries	2 layers of elongated cells	2–3 layers of cells
	Margins of leaf blade	Elliptic-patelliform	elevated nectaries		1–2 layers of cells
*P*. *kermesina* Link & Otto	Petiole	Obconical long-stipitate	elevated nectaries	1–3 layers of elongated cells	2–3 layers of cells
	Margins of leaf blade	Obconical	elevated nectaries		
*P*. *laurifolia* L.	Petiole	Ellipsoid	elevated nectaries	1–2 layers of elongated cells	4–5 layers of cells
*P*. *ligularis* Juss.	Petiole	Obconical asymmetric long-stipitate	elevated nectaries	1–3 layers of elongated cells	1–3 layers of cells
*P*. *maliformis* L.	Petiole	Spheroidal	elevated nectaries	2–4 layers of short cells	2–4 layers of cells
*P*. *miersii* Mart.	Petiole	Obconical	elevated nectaries	1–2 layers of elongated cells	2–4 layers of cells
	Margins of leaf blade	Lenticular	elevated nectaries		1–2 layers of cells
*P*. *odontophylla* Harms ex Glaz	Petiole	Ellipsoid	elevated nectaries	3–4 layers of elongated cells	3–4 layers of cells
	Margins of leaf blade	Concave ocellus	flattened nectaries	2–4 layers of elongated cells	1–2 layers of cells
*P*. *racemosa* Brot.	Petiole	Ellipsoid	elevated nectaries	1 layer of elongated cells	1–3 layers of cells
*P*. *serratodigitata* L.	Petiole	Cotyliform	elevated nectaries	1–2 layers of elongated cells	2–3 layers of cells
	Margins of leaf blade	Elliptic-lenticular to semi-spheroid	elevated nectaries	3–4 layers of short cells	
*P*. *setacea* DC.	Petiole	Speroidal	elevated nectaries	2–3 layers of elongated cells	2–5 layers of cells
*P*. *sidifolia* M. Roem.	Petiole	Obconical short-stipitate	elevated nectaries	2–4 layers of short cells	2 layers of cells
	Margins of leaf blade	Lenticular to elliptic-lenticular	elevated nectaries		
*P*. *sublanceolata* (Killip) MacDougal	Petiole	Clavate	resin glands	1 layer of elongated cells	
	Dispersed on both surfaces of leaf blade	Capitate long-stipitate	resin glands		-
	Margins of leaf blade	Clavate	resin glands		
*P*. *subrotunda* Mast.	Petiole	Obconical	elevated nectaries	1–2 layers of elongated cells	2 layers of cells
	Margins of leaf blade	Lenticular	elevated nectaries	1–3 layers of elongated cells	
*P*. *umbilicata* (Griseb.) Harms	Margins of leaf blade	Elliptic-lenticular	elevated nectaries	1–2 layers of elongated cells	2 layers of cells
*P*. *villosa* Vell.	Petiole	Terete	resin glands	1 layer of elongated cells	-
	Dispersed on both surfaces of leaf blade				
*P*. *watsoniana* Mast.	Petiole	Obconical short-stipitate	elevated nectaries	2–3 layers of elongated cells	2 layers of cells
	Margins of leaf blade	Semi-spheroid	elevated nectaries	1–2 layers of elongated cells	1 layer of cells

(–) No differentiation into secretory parenchyma.

In order to structurally characterize the glands, samples were analyzed through scanning electron microscopy (SEM) and the shape definition was based on the descriptions for solid shapes by Radford *et al*. [22] and the terminology followed Harris & Harris [23]. Samples were fixed in a 50% solution of formaldehyde-ethanol-acetic acid (FAA 50) for 24 hours [24], dehydrated in ascending ethanol series, and then submitted to critical point drying with carbonic gas (CPD 030, Balzer). After drying, samples were mounted in metal stubs and metalized with gold [25]. The analysis was carried out using a QUANTA 250 (FEI COMPANY) scanning electron microscope at the Laboratory for Electron Microscopy of the Santa Cruz State University (UESC, Ilhéus—BA) and using a Zeiss DSM 940 scanning electron microscope (Zeiss, Oberkochen, Germany) at the Institute of Biosciences, University of São Paulo (IB/USP).

For the anatomical analysis, samples of petiolar and laminar glands were fixed in FAA 50 for 24 hours [24] and subsequently stored in a 70% ethanol solution. The material was dehydrated in a butanol series [26] and embedded in Paraplast (Leica Microsystems, Heidelberg, Germany). Longitudinal anatomical sections of the glands varying from 8 to 12 μm thick were cut in a Reihertt-Jung Auto Cut 2040 rotatory microtome and mounted on permanent slides. Material was double stained with a 1% safranin solution in 50% ethanol and a 1% astra blue solution, and mounted on permanent slides with Canada balsam [27]. Acquisition of photographic data was carried out by using a Leica DMLB microscope coupled to a Leica DFC 310FX camera and by using the IM50 software at the Plant Anatomy Laboratory (IB/USP).

In order to analyze the presence of sugars in the secretion of foliar glands, the glucose strip test was used (Inlab Diagnóstica–Alamar Tecno Científica Ltda.) for the species cultivated at the Institute of Biosciences, University of São Paulo. The same method was used for the species *Passiflora sublanceolata* (Killip) MacDougal cultivated in a *Passiflora* active germplasm bank at UESC (BAG-Passifloras).

## Results

In the studied species of *Passiflora*, glands were found in the petioles (petiolar glands) in pairs or dispersed over the petiole at the margin of the leaf blade (marginal glands), on the abaxial face of the leaf blade and/or dispersed on both sides of the foliar blade (laminar glands). In the same species, glands could be seen in more than one region, and the most common combination observed was the presence of petiolar and marginal glands on the same leaf (Table 1).

**Fig 1:**
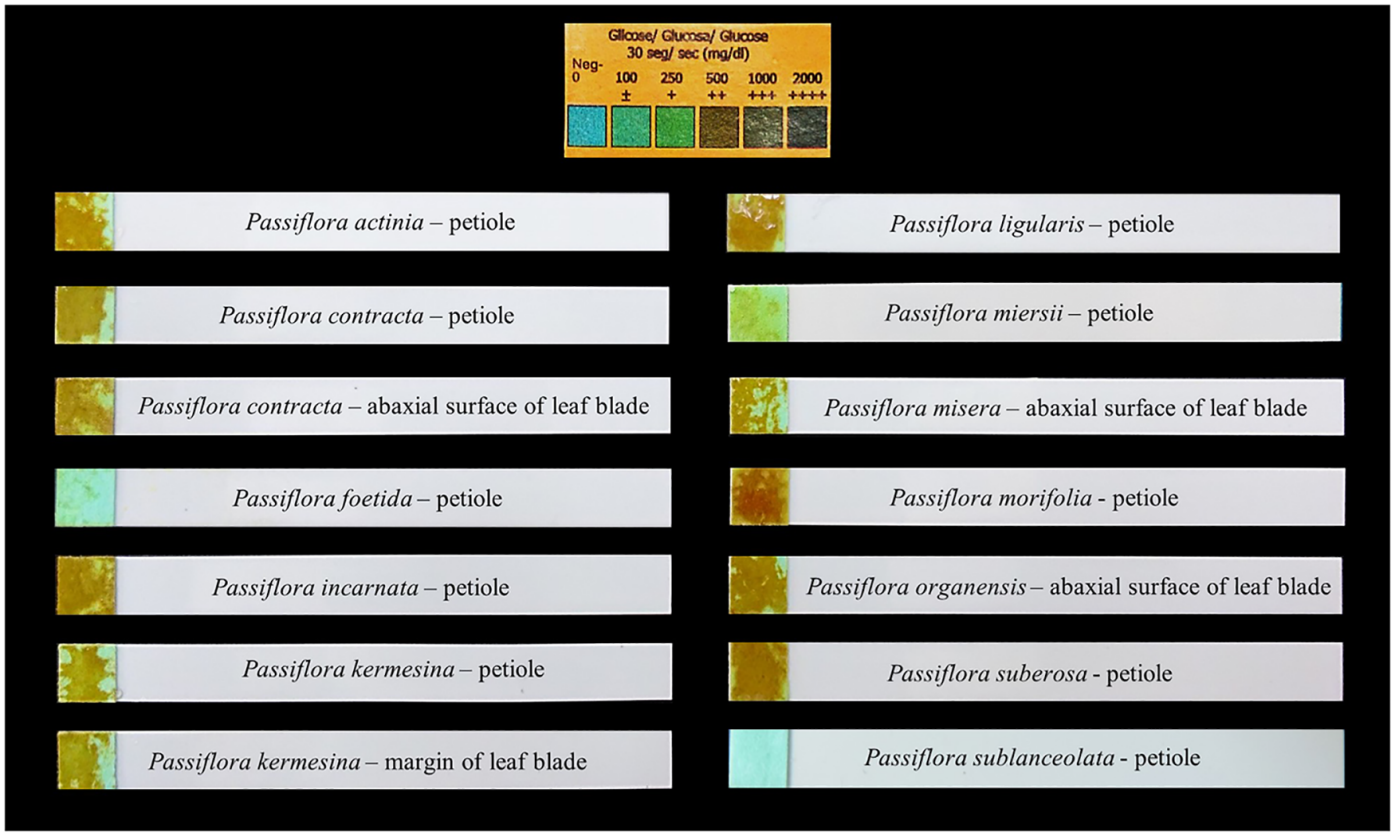
Glucose strip tests of glands in leaves of *Passiflora* L. Positive results are shown for extrafloral nectaries located on petiole of *Passiflora actinia* Hook, petiole and abaxial surface of leaf blade of *P*. *contracta* Vitta, petiole of *P*. *incarnata* L., petiole and margin of leaf blade of *P*. *kermesina* Link & Otto, petiole of *P*. *ligularis* Juss., petiole of *P*. *miersii* Mart., abaxial surface of leaf blade of *P*. *misera* Kunth, petiole of *P*. *morifolia* Mast., abaxial surface of leaf blade of *P*. *organensis* Gardner and petiole of *P*. *suberosa* L. Negative results are shown for glands of *P*. *foetida* L. and *P*. *sublanceolata* (Killip) MacDougal.

**Fig 2:**
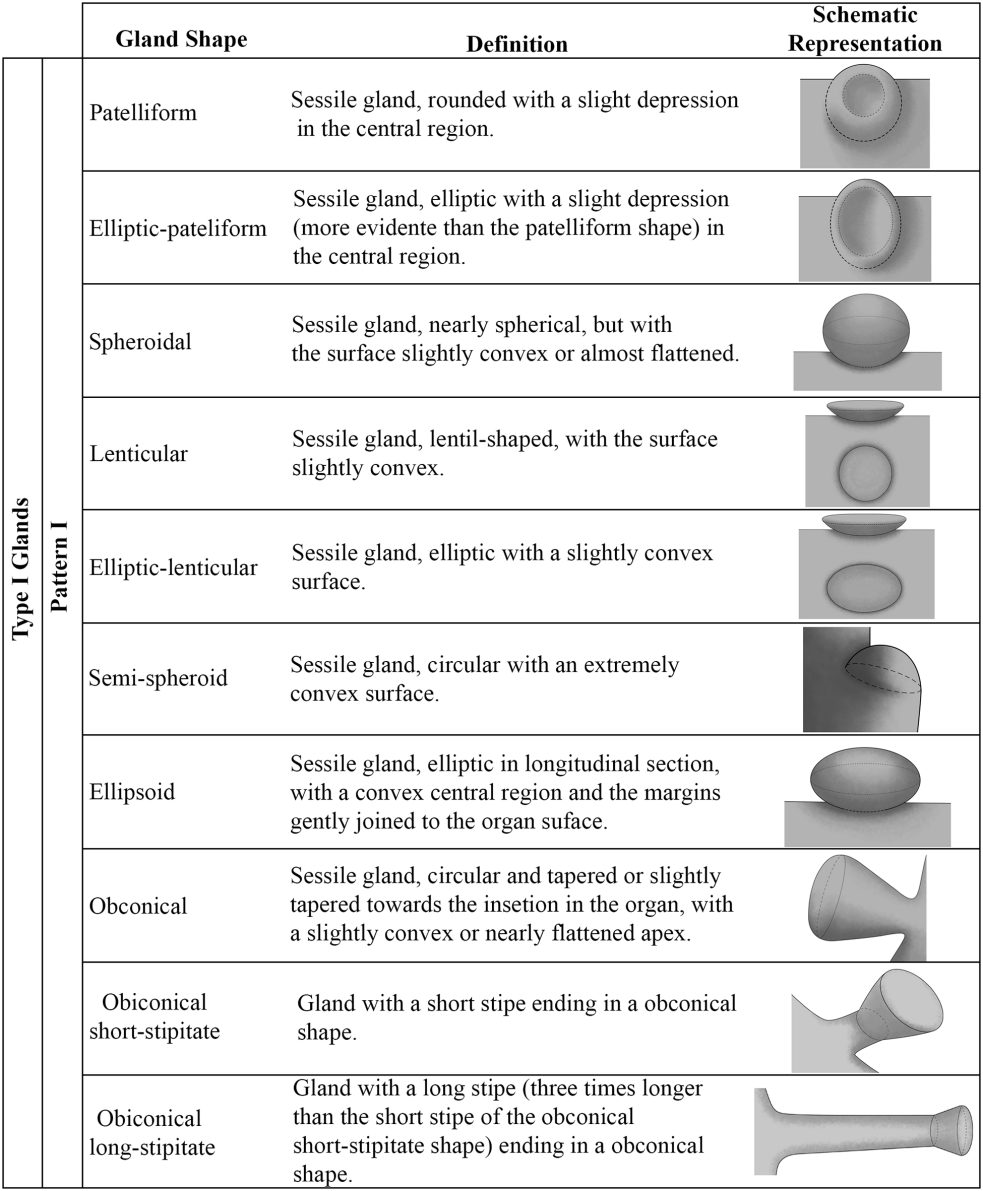
Schematic representation of the gland shapes in *Passiflora* L. Nomenclature used for the different shapes of type I glands, as well as a description and a schematic representative of each one. Illustrations by Yasmin Vidal Hirao.

**Fig 3:**
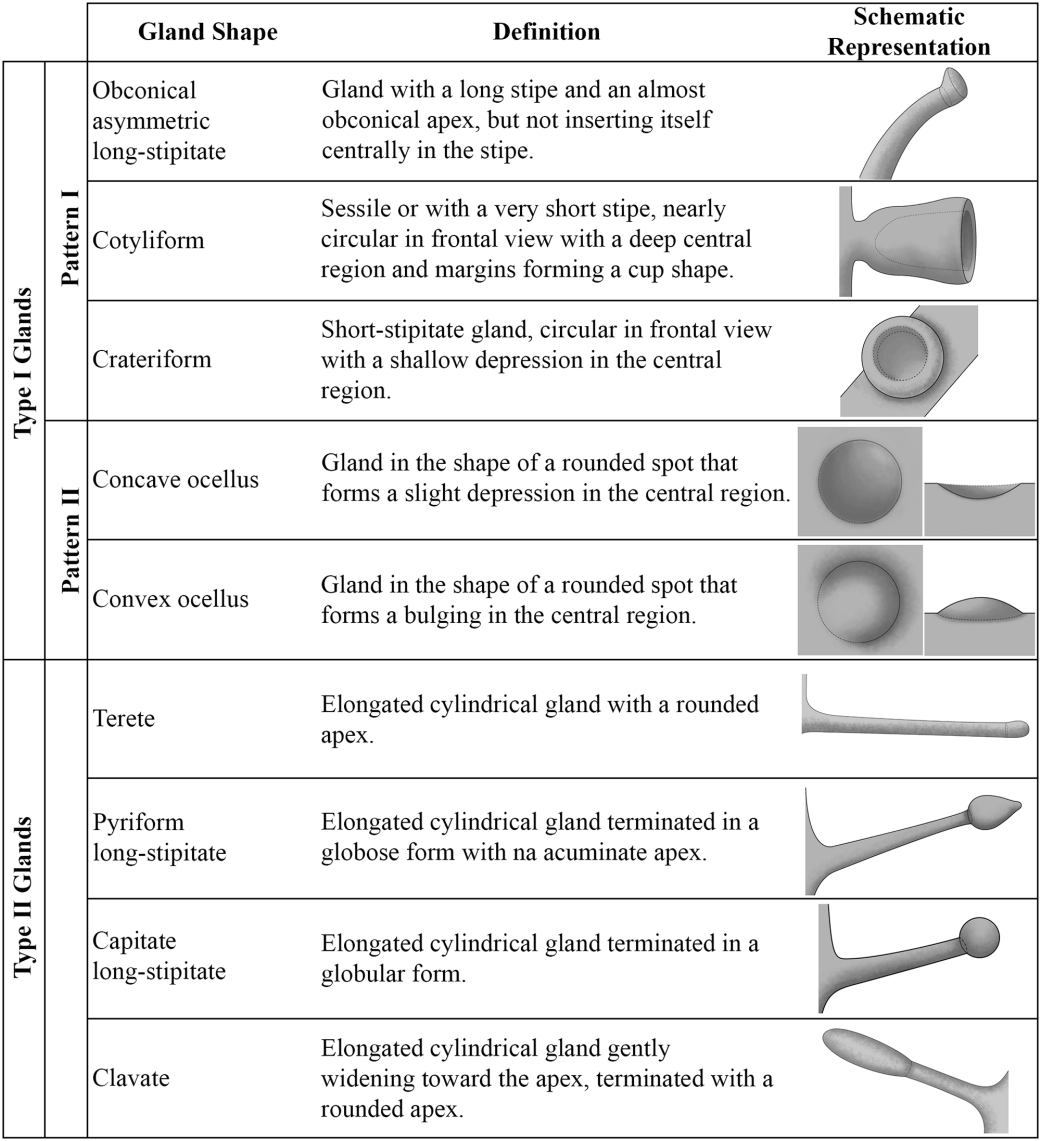
Continuation of Fig 2. Nomenclature used for the different shapes of type I and type II glands, as well as a description and a schematic representative of each one. Illustrations by Yasmin Vidal Hirao.

Based on the results of morphological, anatomical and glucose strip tests (Figs 1–3), the foliar secretory glands in *Passiflora* could be grouped into two categories: Type I glands, either elevated or flattened; and Type II glands, which are elevated (Table 1).

**Type I Glands**–These leaf glands have a sugar content high enough to be detected by the glucose strip test, and as such, they may be defined as nectaries (Fig 1). Nectaries are anatomically formed by palisade secretory epidermis, which is usually multi-layered, followed by secretory parenchymal cells formed by juxtaposed cells with dense content and filled by subsecretory parenchyma cells with large cells in a loose arrangement. Two morphological patterns are described. Pattern I is characterized by well-structured glands with evident projection in relation to the foliar tissue. Pattern II is characterized by glands with tissue closely pressed against the leaf blade tissue, in which the projection is not very evident.

**Fig 4:**
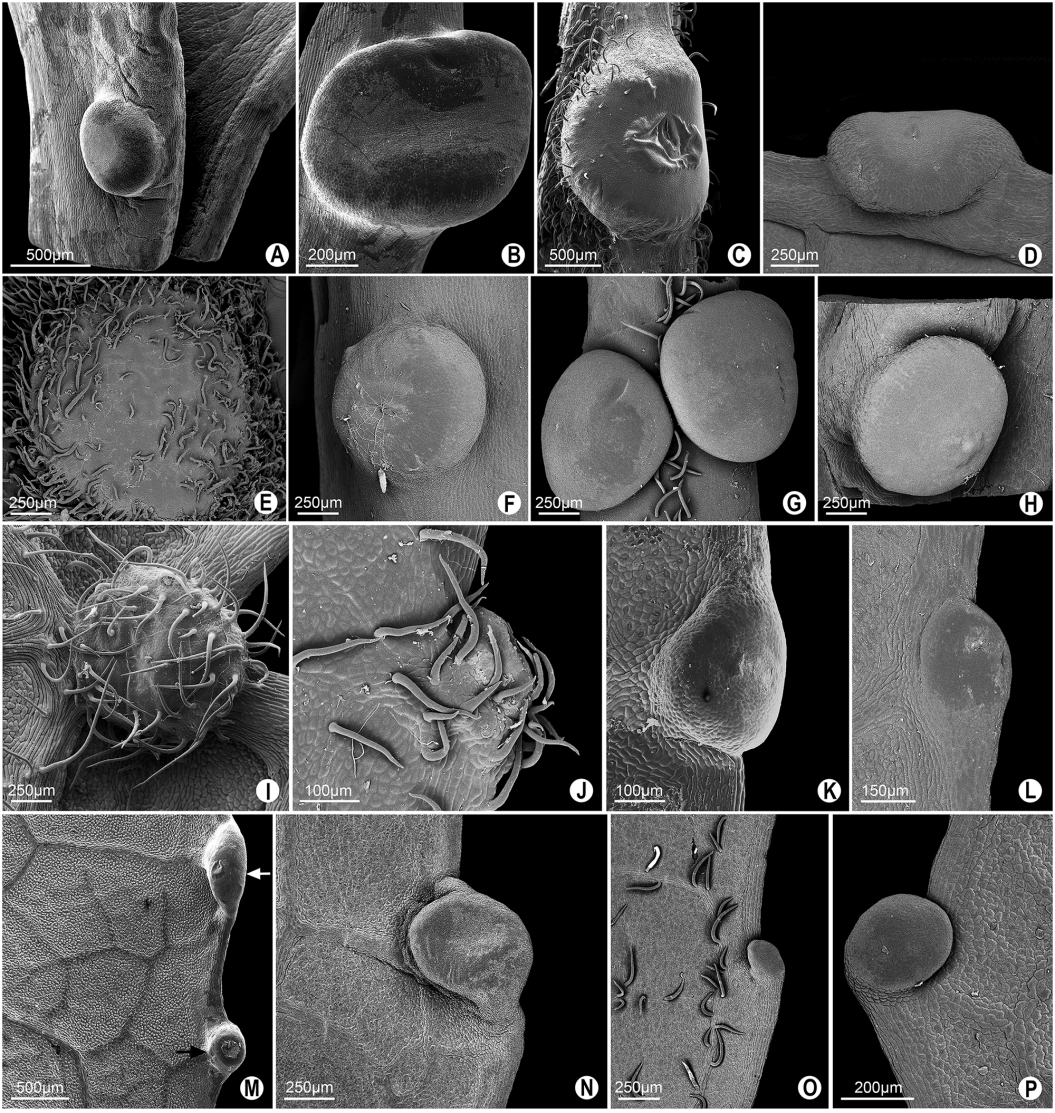
SEM image of the different glands shapes defined as extrafloral nectaries in leaves of *Passiflora* L. **A**. Patelliform gland in the petiolule of *P*. *deidamioides*. Petiolar glands (B, C and E-H). Marginal glands of the leaf blade (D, J-P). **B-D**. Elliptic-patelliform gland in *P*. *deidamioides* and *P*. *incarnata* (C and D). **E-H**. Spheroidal glands of *P*. *coccinea*, *P*. *galbana*, *P*. *maliformis* and *P*. *setacea*, respectively. **I-L**. Lenticular gland in *P*. *coccinea* (abaxial surface of leaf blade), *P*. *gardneri*, *P*. *miersii* and *P*. *subrotunda*, respectively. **M**. Lenticular gland (black arrow) and elliptic-lenticular gland (white arrow) in *P*. *sidifolia*. **N-P**. Elliptic-lenticular glands in *P*. *eichleriana*, *P*. *haematostigma* and *P*. *umbilicata*, respectively.

In **pattern I**, several forms can be grouped (Figs 2 and 3). In *Passiflora deidamioides*, the glands found in the petiolule are patelliform, i.e., sessile, rounded, and with a slight depression in the central region (Fig 4A). On the other hand, the glands of the petiole are more elongated with an elliptic-patelliform shape, and a more evident depression in the central region (Fig 4B). This elliptic-patelliform shape also occurs in petiolar and marginal glands of *P*. *incarnata* (Fig 4C and 4D.

The petiolar glands of *Passiflora coccinea*, *P*. *galbana*, *P*. *maliformis* and *P*. *setacea* are spheroidal, with a flattened or slightly convex surface (Fig 4E–4H). The glands on the abaxial surface of leaf blade of *P*. *coccinea* and the marginal glands of *P*. *gardneri*, *P*. *mierssi*, *P*. *sidifolia* and *P*. *subrotunda* are lenticular ones (Fig 4I–4M). In some glands, a distension of the cuticle occurs, probably owing to the accumulation of exudate in the subcuticular space (Fig 4H and 4K).

**Fig 5:**
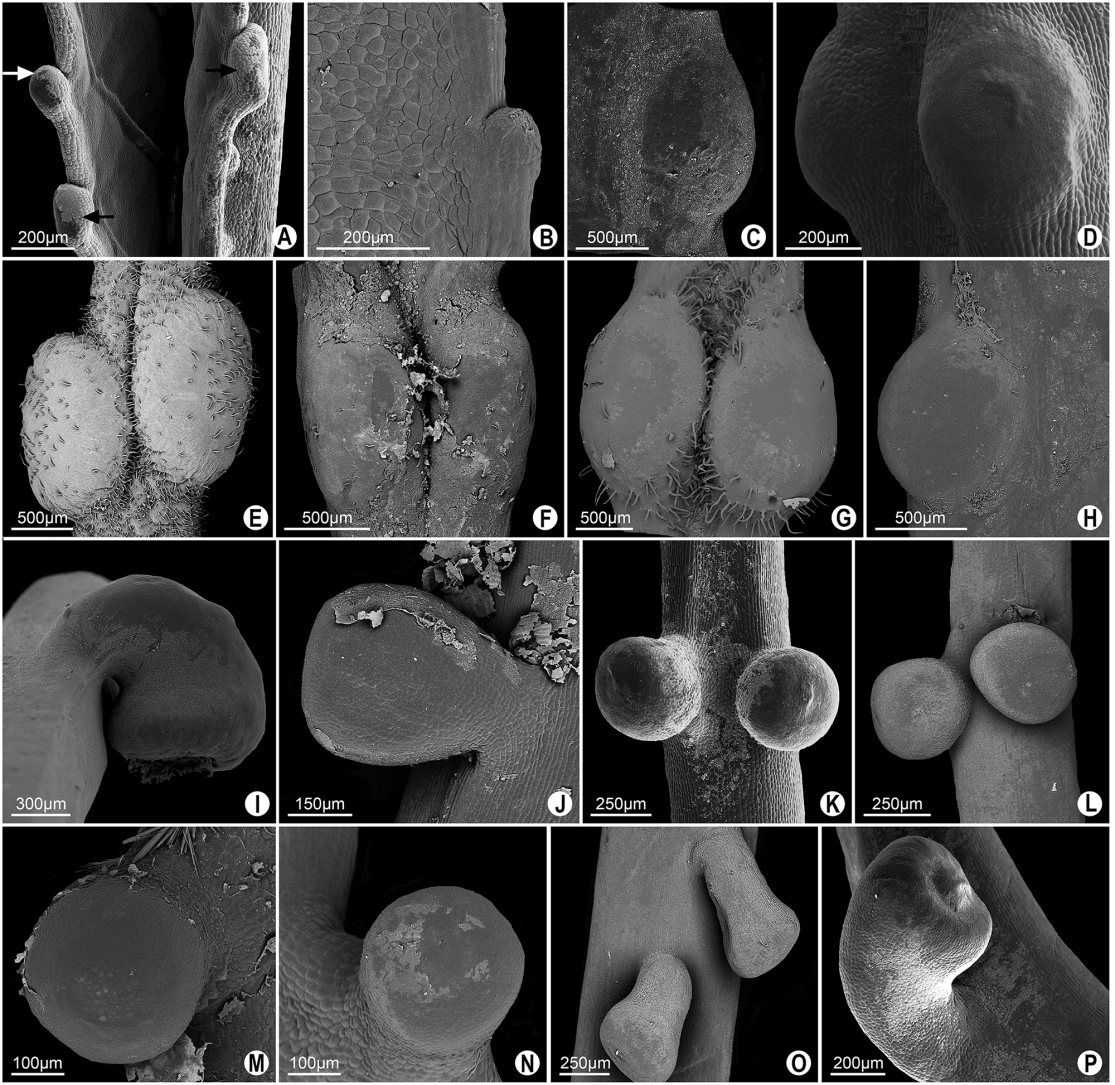
Continuation of Fig 4. Marginal glands (A, B, M, N). Petiolar glands (C-L, O, P). **A**. Elliptic-lenticular glands (black arrows) and semi-spheroid gland (white arrow) in *P*. *serratodigitata*. **B**. Semi-spheroid gland in *P*. *watsoniana*. **C-H**. Ellipsoid glands in *P*. *ambigua*, *P*. *contracta*, *P*. *haematostigma*, *P*. *laurifolia*, *P*. *odontophylla* and *P*. *racemosa*, respectively. **I-N**. Obconic glands in *Passiflora actinia* (note a curvature towards the abaxial region of the petiole), *P*. *elegans*, *P*. *miersii*, *P*. *subrotunda*, *P*. *elegans* (frontal view) and *P*. *kermesina* (frontal view), respectively. **O-P**. Obconic short-stipitate glands in *P*. *eichleriana*, *P*. *sidifolia* (also curved towards the abaxial region of the petiole), respectively.

In *Passiflora sidifolia* (Fig 4M), the marginal glands have different shapes. Some glands are more elongated than those previously described, and thus termed as elliptic-lenticular glands. This form also occurs in the marginal glands of *P*. *eichleriana*, *P*. *haematostigma*, *P*. *umbilicata* and *P*. *serratodigitata* (Figs 4N–4P and 5A). Besides having elliptic-lenticular glands in the leaf margins, *P*. *serratodigitata* (Fig 5A) may also have semi-spheroid glands in this region. This shape is also found in the marginal glands of *P*. *watsoniana* (Fig 5B). These glands are sessile, circular, and widely convex.

A slightly different shape from those previously mentioned is the ellipsoid one. The ellipsoid shape occurs in the petiolar glands of *P*. *ambigua*, *P*. *contracta*, *P*. *haematostigma*, *P*. *laurifolia*, *P*. *odontophylla* and *P*. *racemosa* (Fig 5C–5H). These glands are sessile, more elongated in their longitudinal axis, have a convex central region, and their margins join gently to the petiole. In *P*. *haematostigma* and *P*. *odontophylla* (Fig 5E and 5G) non-glandular trichomes were observed in the nectariferous epidermis, most abundantly in *P*. *haematostigma*.

The petiolar glands of *P*. *actinia*, *P*. *elegans*, *P*. *miersii* and *P*. *subrotunda* (Fig 5I–5L), as well as the marginal glands of *P*. *elegans* and *P*. *kermesina* (Fig 5M and 5N, in frontal view), are sessile and display an obconical shape, being circular and slightly tapered towards the insertion in the petiole, with the apex flattened or slightly convex. In *P*. *actinia*, a curvature is seen towards the abaxial region of the petiole (Fig 5I).

**Fig 6:**
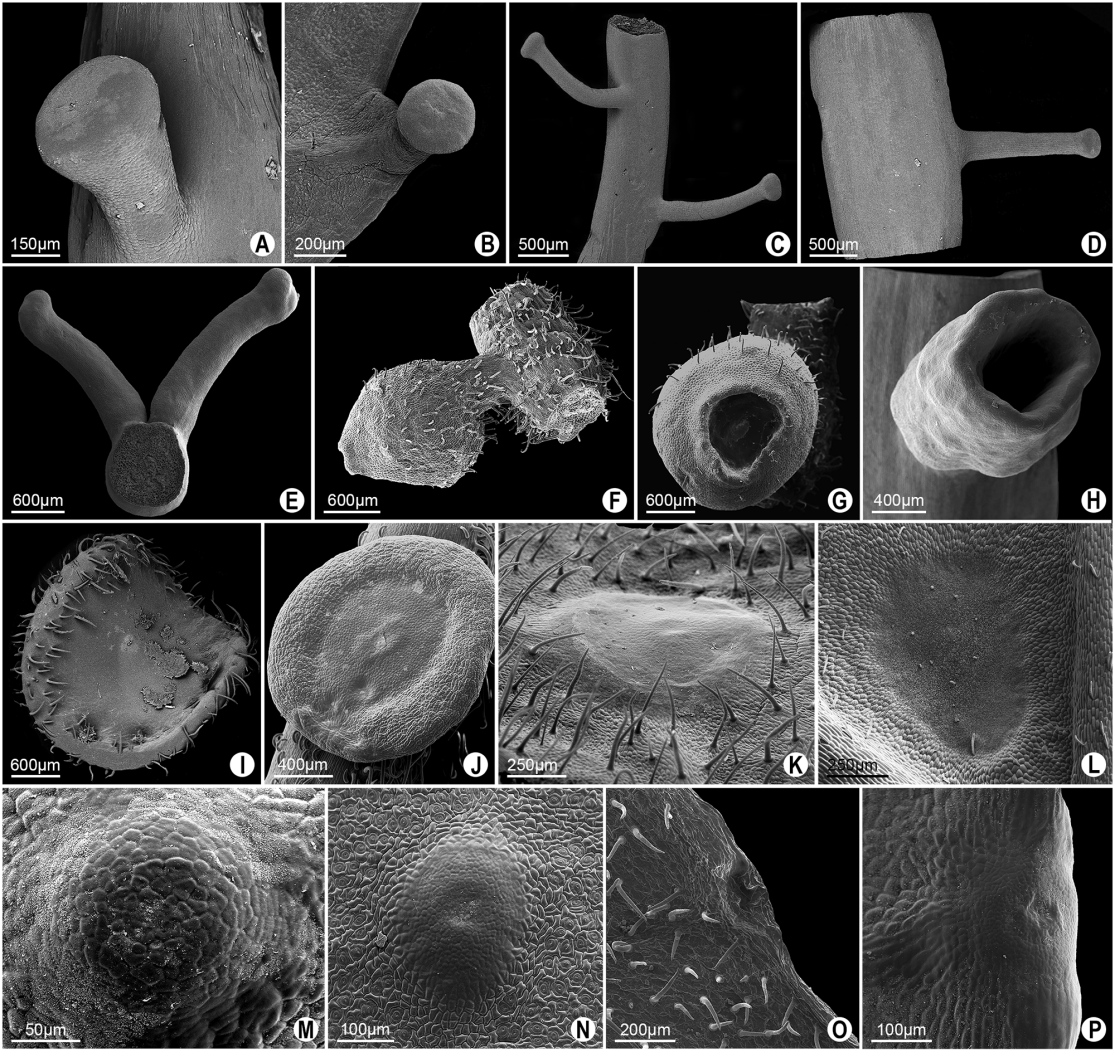
Continuation of Fig 5. Petiolar glands (A, C-J). Glands on the abaxial surface of leaf blade (K-N). **A-B**. Obconic short-stipitate glands of *P*. watsoniana and *P*. *edmundoi*, respectively. **C-D**. Obconic long-stipitate glands of *P*. *edmundoi* and *P*. *kermesina*. **E**. Asymmetric long-stipitate glands of *P*. *ligularis*. **F-H**. Cotyliform glands in *P*. *morifolia* (F in lateral view and G in frontal view) and *P*. *serratodigitata* (H). **I-J**. Crateriform glands in *P*. *ferruginea* and *P*. *suberosa*, respectively. **K-L**. Detail of concave ocellus glands in *P*. *ferruginea* and *P*. *misera*, respectively. **M-N**. Detail of convex ocellus glands in *P*. *organensis* and *P*. *contracta*, respectively. **O-P**. Detail of concave ocellus glands on margin of leaf blade in *P*. *odontophylla* and *P*. *galbana*, respectively.

Similarly to the previous form, we have identified obconical short-stipitate glands in which a short stipe ends in an obconical shape. These glands occur in the petiole of *P*. *eichleriana* and *P*. *sidifolia*, in which they make a curvature towards the abaxial region of the petiole, as well as in *P*. *watsoniana* (Fig 5O, 5P and 6A) and in the leaf margin of *P*. *edmundoi* (Fig 6B).

On the petiole of *Passiflora edmundoi* and *P*. *kermesina*, an obconical long-stipitate gland is observed with a stipe three times longer than that of short-stipitate glands (Fig 6C and 6D). A variation of this type of gland is noted in *P*. *ligularis*, in which a long stipe ends in a nearly obconical shape, but without inserting itself centrally in the stipe. This gland was determined to be obconical asymmetric long-stipitate (Fig 6E).

In *Passiflora morifolia* (Fig 6F and 6G) and *P*. *serratodigitata* (Fig 6H), the petiolar glands show a very short stipe ending in a cotyliform shape. Here, the central region is deep, and the margins of glands form a cup shape.

The crateriform shape occurs in the short-stipitate petiolar glands, as observed in *P*. *ferruginea* and *P*. *suberosa*. These glands are circular and flat, with a shallow depression in the central region (Fig 6I and 6J).

Among the **nectaries that follow pattern II**, the glands on abaxial surface of leaf blade have the shape of a rounded spot, which may form a slight depression, termed as concave ocellus, or a slight bulging, termed as convex ocellus (Fig 2). In *P*. *ferruginea*, *P*. *misera*, and *P*. *organesis*, a pair of ocellus concave glands (Fig 6K and 6L) are usually observed in the region among the main vascular bundles at the base of the foliar blade, as well as convex ocellus glands (Fig 6M) dispersed in the foliar blade. In *P*. *contracta*, the glands on abaxial surface of leaf blade are convex ocellus only (Fig 6N). Slight concave ocellus glands also occur at the margin of the leaf blade in *P*. *galbana* and *P*. *odontophylla* (Fig 6O and 6P). These glands are minute and hardly recognizable to the naked eye.

**Fig 7:**
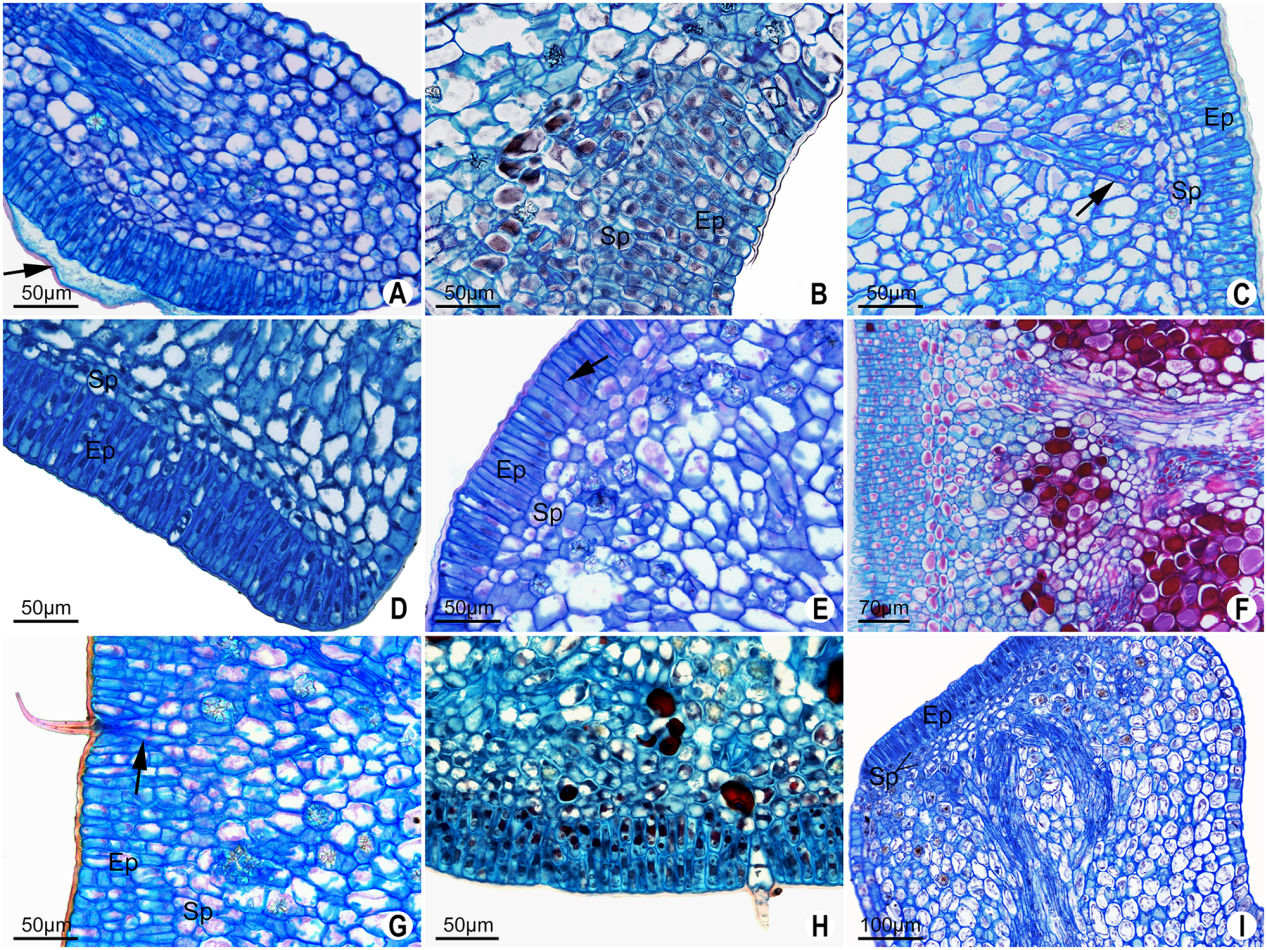
Longitudinal sections of extrafloral nectaries (EFN) in *Passiflora* L. Marginal of the leaf blade EFN (A). Petiolar EFN (B-G, I). Abaxial surface of leaf blade EFN (H). **A**. *P*. *incarnata*, note exudate in the subcuticular space and nuclei centralized in secretory epidermal cell. **B**. *P*. *suberosa* with short secretory epidermal cells. **C**. *P*. *incarnata* with elongated secretory epidermal cells and secretory parenchyma with flattened cells, note the vascular bundle ending with phloem cells (arrow). **D**. *P*. *eichleriana*, elongated secretory epidermal cells and secretory parenchyma with flattened cells. **E**. *P*. *racemosa* showing elongated secretory epidermal cells, note regions with two layers of cells (arrow). **F**. *P*. *deidamioides*, showing distinctive secretory parenchyma cells and subsecretory parenchyma with vascular bundles. **G**. *P*. *haematostigma* with unicellular trichome and extensions of the subepidermal parenchyma (arrow). **H**. Multicellular trichome in *P*. *coccinea*. **I**. *P*. *gabana* with elongated secretory epidermal cells and vascular bundles ending with phloem cells in the subsecretory parenchyma. Ep = epidermis; Sp = secretory parenchyma.

The secretory epidermis is usually located in the central region of the glands. In some cases, it is restricted to either one of the sides, occupying a region that is proportionally small relative to the total size of the glandular projection (S1–S4 Figs). A subcuticular space is formed by distension of the cuticle caused by the accumulation of exudate (Fig 7A). Epidermal cells are generally elongated in the anticlinal direction, but they may vary from short to elongated (Fig 7 and Table 1). The nuclei are relatively large, usually centralized (Fig 7D), and the cell content is dense. The number of cells layers constituting the secretory epidermis can be variable in the same species and even in the same gland (Table 1). In *P*. *racemosa*, a predominant single layer of elongated cells is observed to form the secretory epidermis. However, regions with more than one layer are often found in the same nectary (Fig 7E). The non-secretory epidermis only shows one layer of cells with a slightly evident nucleus and a large vacuole.

Multicellular and unicellular non-glandular trichomes can be found in the secretory epidermis of *P*. *coccinea* and *P*. *haematostigma* (Fig 7G). Extensions of the subepidermal parenchyma occur in the regions underlying the base of the trichomes.

**Fig 8:**
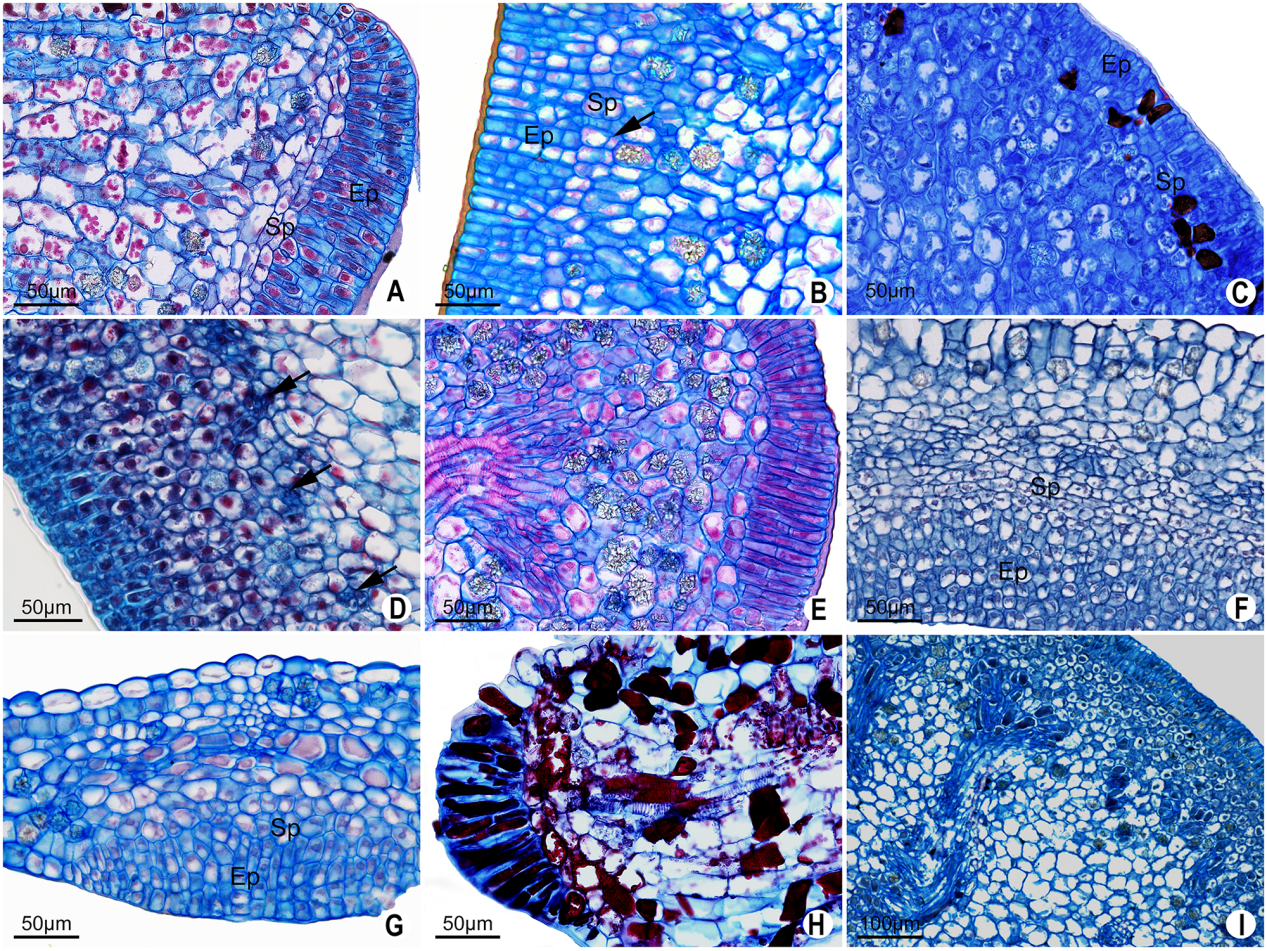
Continuation of Fig 7. Petiolar EFN (A-D, I). EFN of the abaxial surface of leaf blade (F-G). EFN on the margin of leaf blade (H). **A**. *P*. *kermesina* with elongated secretory epidermal cells and secretory parenchyma with flattened cells. **B**. *P*. *haematostigma* with secretory parenchyma slightly smaller and more juxtaposed than the layers of the subsecretory parenchyma. Note the periclinal divisions on the secretory parenchyma (arrow). **C**. *P*. *laurifolia* with unclear limits between secretory parenchyma and no-secreting parenchyma. In the secretory parenchyma some cells show a dense content and are smaller than the cells of the subsecretory parenchyma. **D**. *P*. *ferruginea* showing extensive secretory parenchyma and vascular endings formed by phloem cells (arrow). **E**. *P*. *subrotunda* showing elongated secretory epidermal cells and druses on the subsecretory parenchyma. **F-G**. Short secretory epidermal cells in *P*. *misera* (concave ocellus) and *P*. *organensis* (convex ocellus), respectively. **H**. *P*. *gardneri* with vascular ending composed of both phloem and xylem cells. **I**. *P*. *setacea* showing vascularization with ramifications. Ep = epidermis, Sp = secretory parenchyma.

Internal to epidermis, the secretory parenchyma is usually formed by small cells, which can be flattened (Figs 7C, 7D, 7F and 7I and 8A), and have an evident nucleus and dense content. In *P*. *haematostigma* and *P*. *laurifolia*, the border between this region and the cells of the subsecretory parenchyma is unclear. In *P*. *haematostigma* the cells are slightly smaller and more juxtaposed than the innermost layers, and they are apparently derived from the hypodermis by periclinal divisions (Fig 8B). In *P*. *laurifolia*, the secretory parenchyma cells are smaller than those of subsecretory parenchyma and with a dense cytoplasma content (Fig 8C).

The secretory parenchyma does not show druses or vascular endings in most species. Nevertheless, vascular endings formed only by phloem occur in inner layers of the secretory parenchyma in *Passiflora ferruginea* and *P*. *suberosa* (Figs 8D and 7B). Druses do occur in *P*. *ferruginea*, *P*. *morifolia*, *P*. *setacea*, *P*. *incarnata*, *P*. *laurifolia*, *P*. *maliformis*, *P*. *odontophylla* (petiolar gland) and *P*. *racemosa*.

The innermost region of the glands is formed by non-secreting parenchyma (subsecretory), vascular bundles and idioblasts with druses (Figs 7 and 8). This is a highly variable region in that it can form glandular projections (S1–S4 Figs). Generally, the extension of the subsecretory parenchyma of marginal glands is limited to a few layers of cells. In this regard, the marginal gland of *P*. *watsoniana* is particularly short. Extension of the subsecretory parenchyma in petiolar glands is particularly notable in *P*. *ligularis* (S3D Fig). In glands on the abaxial surface of leaf blade, the subsecretory parenchyma replaces the palisade parenchyma present in the rest of the foliar blade, thus forming a very distinct region of parenchyma with rounded cells and large vacuoles occupying most of the cells (Fig 8F and 8G).

Vascularization of the glands occurs up to the region of the subsecretory parenchyma and consists of collateral bundles. In most species, the endings only consist of phloem cells (Fig 7C and 7I), but in *P*. *gardneri* vascular endings are composed of both phloem and xylem cells (Fig 8H).

In petiolar glands, the vascularization can show many ramifications (Fig 8I) or a single bundle such as that found in the marginal glands (Fig 7A). In the glands of abaxial surface of leaf blade, general vascularization runs parallel to the leaf surface, with the phloem tissue closer to the secretory tissue (Fig 8F and 8G).

**Fig 9:**
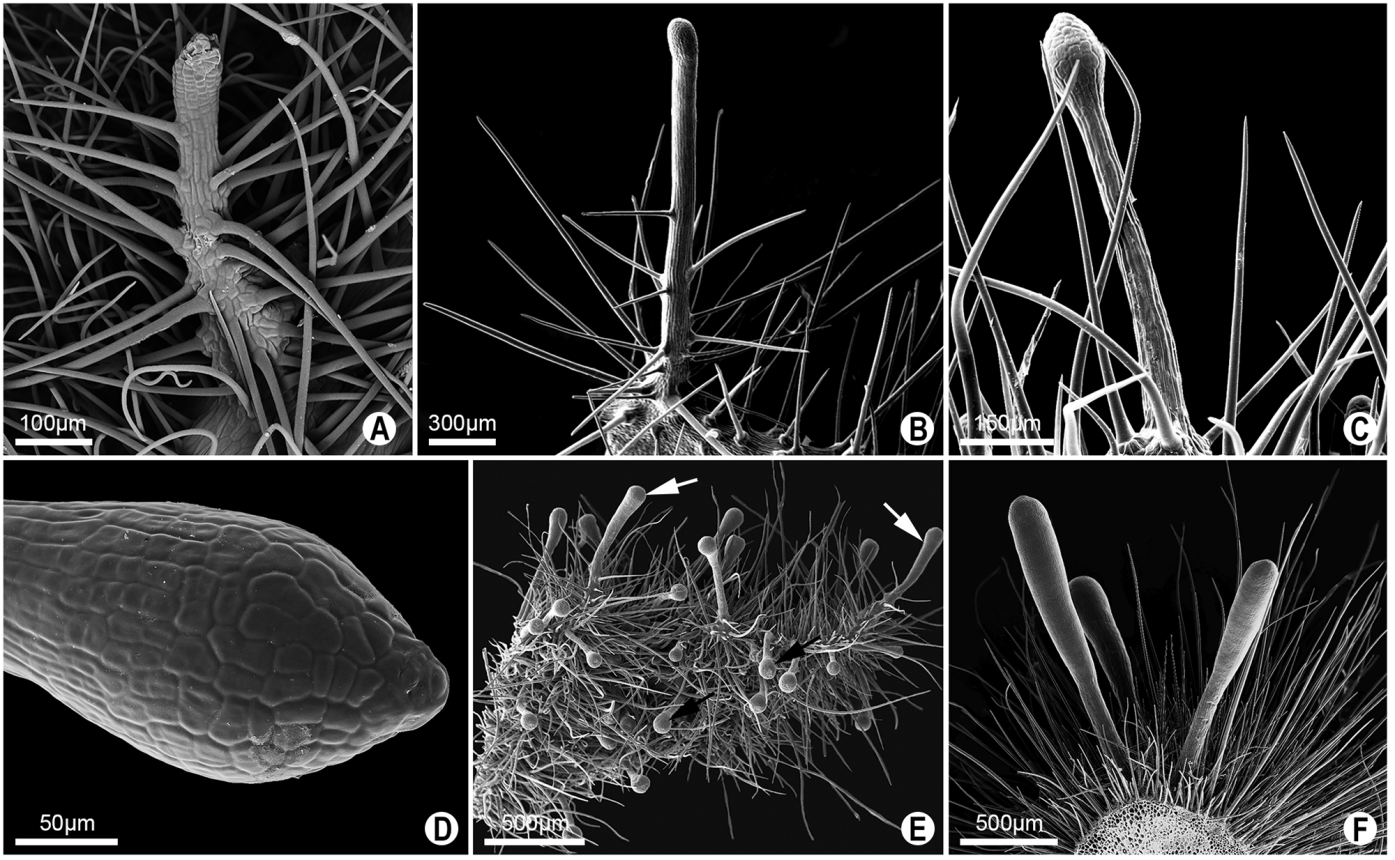
SEM image of type II glands in leaves of *Passiflora* L. **A-B**. Terete gland with trichomes along the stipe in *P*. *arida* and *P*. *villosa*, respectively. **C-D**. Pyriforme long-stipitate gland on the petiole in *P*. *foetida*. Detail of capitate apical region (D). **E**. Capitate long-stipitate (black arrow) and clavate (white arrow) glands on the lamina of *P*. *sublanceolata*. **F**. Clavate glands on the petiole of *P*. *sublanceolata*.

**Fig 10:**
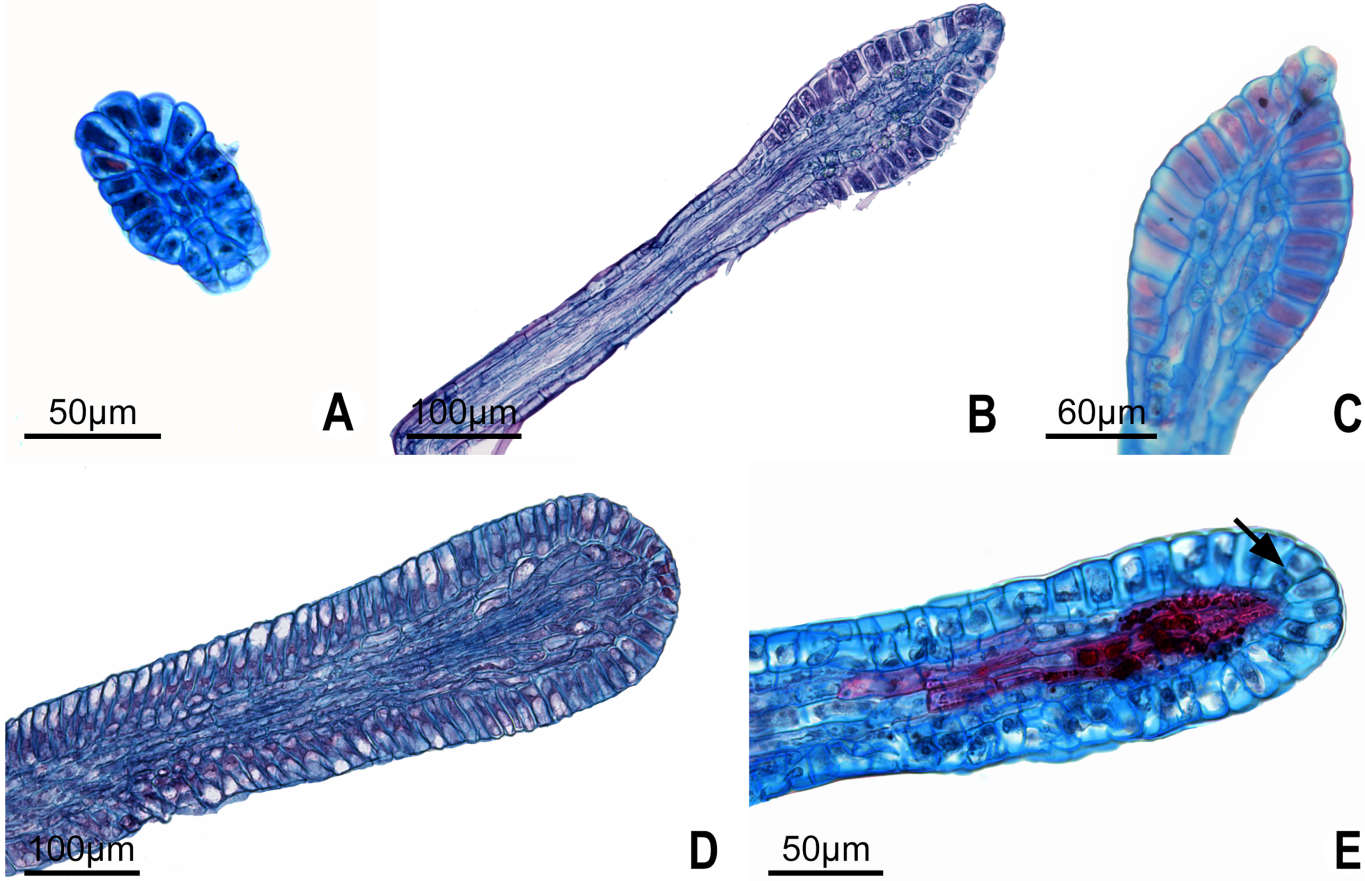
Longitudinal sections of type II glands in *Passiflora* L. In all cases, the secretory region is restricted to elongated epidermal cells. **A-B**. Petiolar gland of *P*. *arida* and *P*. *foetida*, respectively. **C**. Detail of elongated epidermal cells of a laminar gland in *P*. *foetida*. **D**. Petiolar gland in *P*. *sublanceolata*. **E**. *P*. *villosa* with lignified parenchyma cells, note the dividing secretory epidermal cells (arrow).

**Gland type II**- A single pattern was observed for this glandular type, which did not show a positive reaction to the glucose strip test. These glands may be found in the petiole or across the entire foliar blade, showing a minute size, and thus being easily mistaken for trichomes (Fig 9). Anatomically, these glands have secretory epidermis with a single layer of anticlinally elongated cells. No distinction can be observed between secretory and non-secreting parenchyma (Fig 10).

In *P*. *arida* and *P*. *villosa*, the petiolar and laminar glands found on both sides and on the margin of the leaves are elongated, with trichomes along the stipe and with a rounded apex (Fig 9A and 9B). These are termed “terete” glands (Fig 3).

On the other hand, the petiolar and laminar glands of *Passiflora foetida*, termed pyriform long-stipitate, do not show trichomes in their long stipes, but the termination is globose with an acuminate apex (Figs 3 and 9C). Similar to these glands are the non-marginal laminar glands of *P*. *sublanceolata*, which differ in the globular, non-pyriform termination observed in the previous description. They may be denominated as capitate long-stipitate (Figs 3 and 9E). Clavate glands at the margins of the leaf blade and in the petiole are found in *Passiflora sublanceolata* (Figs 3, 9E and 9F).

In these species, the glands are elongated with the secretory region restricted to the apex, and formed by palisade secretory epidermis (Fig 10A–10E). The epidermal cells of the stipe region are periclinally elongated, short in the anticlinal direction (Fig 10B), and in *P*. *arida* and *P*. *villosa*, trichomes are found along it. The inner region has few layers of parenchyma cells, some of which have druses (Fig 10C). The vascular system is central, and it has xylem and phloem endings. A gradual tapering in the secretory region of *Passiflora foetida* ends with the two most apical cells (Fig 10C). In *Passiflora sublanceolata*, the petiolar and marginal glands have a broader secretory region that extends almost to the middle part of the gland (Fig 10D). In addition, the subepidermal cells are wider in the petiolar glands of *P*. *sublanceolata* (Fig 10D) which has fewer druses than what is found in *P*. *foetida*. In *Passiflora arida*, the secretory region is more restricted, with shorter secretory cells with thin walls (Fig 10A).

In *Passiflora villosa*, some secretory epidermal cells divide (Fig 10E). However, a multiseriate epidermis does not occur, as it does in species that have nectaries. In the subepidermal layers, lignified parenchyma occurs in some glands (Fig 10E).

## Discussion

Our comparative study has revealed a variety of forms among the extrafloral secretory glands. Therefore, we were able to classify them into two distinct categories. Type I glands can be elevated or flattened, and have a sugar content high enough to be detected by the glucose strip test. These glands are defined as nectaries. Type II glands are elevated, but show no positive reaction to the glucose strip test. With few exceptions, nectar secreting glands are present in *Passiflora* [5, 8], and the presence of EFN in Passifloraceae, as well as their shape, position and number, have been used as an important diagnostic feature for species or groups of species within the family [2–10].

*Passiflora* subgenera show EFN distributed as distinct features. *Astrophea* has two petiolar nectaries near the leaf blade; nectaries are absent on the leaf blade. *Deidamioides* has two petiolar nectaries or inconspicuous nectaries at the margin of the leaf blade; nectaries can also be absent on the leaf blade. *Decaloba* has two (rarely more than four) petiolar nectaries, when present, and leaf blades with nectaries in the shape of spots. *Passiflora* has two to six petiolar nectaries (rarely absent or more than six), and in the foliar blades nectaries may be either absent or present (marginal ones) [1, 2]. *Tetrapathea* has petiolar nectaries, up to two per petiole when present, in ovoid, sub-sessile or crateriform shapes. When present, it has up to eight laminar nectaries that are ovoid and, in some cases, inserted in pairs between the median and primary veins at the base of the leaf blade, or even dispersed along the major veins [3].

Among the analyzed species, we found considerable morphological diversity in the glands of the subgenus *Passiflora*, many species displaying both petiolar and laminar glands in the same specimen. As mentioned in the description of the subgenus, the laminar glands observed in the present study were marginal, with the exception of the glands of the abaxial surface of leaf blade of *P*. *coccinea*. Still, Silva *et al*. [28] reported the occurrence of ocellus EFN on abaxial surface of leaf blade in at least one species of the subgenus *Passiflora*, *P*. *glandulosa* Cav. In the subgenus *Decaloba*, the analyzed species have only one type of gland, either petiolar or dorso-laminar, except for *P*. *ferruginea*, which has petiolar glands and ocellus glands on abaxial surface of leaf blade. Among the species belonging to the subgenus *Deidamioides*, *P*. *deidamioides* show features in common with those described for the group, with two petiolar nectaries, in this case, nectaries are also present in the petiolule. However, for *P*. *contracta*, besides the petiolar nectaries, nectaries in the abaxial surface of leaf blade are also present. Unlike the description for the group, *P*. *haematostigma*, from the subgenus *Astrophea*, has nectaries on the foliar blade in addition to petiolar nectaries.

Among species that show glands defined as EFN and verified through chemical tests for the presence of total carbohydrates [17, 28–30], most of them have glands that fit exclusively into the pattern I, as described in the present work. Since this morphological type is representative of EFN in *Passiflora*, we can denominate such pattern I glands as **elevated nectaries** ("Hochnektarien") according to the classification of Zimmermann [16]. In describing the structure of EFN in *Passiflora* species, other authors, such as Durkee [17] and Silva *et al*. [28], have already classified these glands as elevated nectaries. Analyzing the EFN in *Piriqueta* and *Turnera* (genera currently within Passifloraceae) Gonzales & Ocantos [31] classified most of the nectaries as elevated ones.

In pattern II, the glands that occur on abaxial surface and margin of leaf blade have a circular shape forming an ocellus, and the projection in relation to the leaf tissue is not evident to the naked eye. In this group, we mention *Passiflora contracta*, *P*. *ferruginea*, *P*. *misera* and *P*. *organensis*, two of which have glands exclusively on the abaxial surface of the leaf blade (*P*. *misera* and *P*. *organensis*), while the other two have petiolar glands that fall under morphological pattern I. The marginal glands included in this morphological type are present in only two of the analyzed species, *P*. *galbana* and *P*. *odontophylla*. Since this morphological pattern is also representative among the EFN of *Passiflora*, we classified these glands as **flattened nectaries** ("Flachnektarien") according to Zimmermann [16]. Durkee [17] mentioned the nectaries of the abaxial surface as embedded in the surface of the organ, a category included by Elias [11] to Zimmermann [16] classification, whereas Silva *et al*. [28] classified the nectaries on abaxial surface of leaf blade as pit nectaries according to the Zimmermann [16] classification. Gonzales & Ocantos [31] also placed the EFN of *Piriquetta* Aubl. and *Turnera* L. (Turneraceae, currently Passifloraceae) present on abaxial surface of leaf blade and some marginal glands among the flattened nectaries.

The nomenclature of the different glandular shapes varies among authors. The description of the glands for some species is often mentioned only as sessile or stipitate, or as concave or convex [5, 10, 28, 30].

In this work, we are using nomenclature for gland shape based on the solid form descriptions found in Radford *et al*. [22] and Harris & Harris [23]. This approach facilitates comparative studies, such as ours, through nomenclatural standardization, also contributing to a more accurate and diverse definition of the variety of glandular forms found among the different *Passiflora* species. When analyzing EFN in several species of *Piriquetta* and *Turnera* (Turneraceae, currently Passifloraceae), Gonzales & Ocantos [31] divide the nectaries into four distinct shapes: flat (for nectaries on abaxial surface of leaf blade), as well as globose, hemispherical and cupuliform, which are related to elevated nectaries. However, we chose not to use this classification because it would have limited our ability to distinguish among the glandular shapes found in the species studied here, thus not highlighting the great variety found in *Passiflora*.

Anatomically, the EFN show small variations of the same pattern among the various studied species. All extrafloral glands framed as nectaries are vascularized nectaries according to the definition of Elias [11]. Anatomical analysis reveals a multi-layered secretory epidermis (in palisade), varying in number of layers, followed by two layers of secretory parenchyma cells with dense content. This number is also variable between different species and even within a single nectary. Internally, vascular bundles with phloem endings occur in a subsecretory parenchyma. These characteristics are very similar to those already described for EFN in *Passiflora* species [17, 28–30, 32], despite the differences in nomenclature in some works. Durkee [17] named the nectary epidermis as “secretory tissue”, which the author described as being followed by 2–5 layers of non-secretory tissue (nectary parenchyma) that separates the secretory tissue (nectary epidermis) from the vascular supply.

In *Passiflora* species, the secretory parenchyma contains isodiametric cells that are larger than those of the epidermis, usually more vacuolated with a dense cytoplasm and abundant calcium oxalate crystals [17, 18, 28]. In the species studied here, cells of the secretory parenchyma are generally larger than the secretory epidermal cells, but they may also be flatter, being found with dense vacuolar content and druses in few species, which are more abundant in the subsecretory parenchyma. In some cases, the differentiation between nectary and subsecretory parenchyma is a fine one. In this region, Jáuregui *et al*. [32] report that the cells have a slightly thickened wall and that they are also very compact and almost indistinguishable from the secretory epidermis. In the species studied here the difficulty of distinguishing between the secretory epidermis and the secretory parenchyma can be verified only in some glands on abaxial surface of leaf blade. Through an ontogenetic study of glands present on the abaxial surface of leaf blade, Roth [33] verified that this region originates by periclinal divisions of the subepidermal layer and that anticlinal rows do not form as in the epidermis. In the studied glands, it was possible to detect periclinal divisions of the subepidermal layer, or hypodermis, occurring not only in the glands of abaxial surface of leaf blade, but also in those of the elevated type.

In addition to the presence of EFN, as described by several authors in Passiforaceae, Solereder [15] reported the occurrence of "glandular shaggy hairs" in *Passiflora* and *Malesherbia* (currently Passifloraceae). In the genus *Passiflora* the author reported that these "trichomes" are restricted to some species like *Passiflora clathrata* Mast., *P*. *foetida*, *P*. *lepidota* Mast. and *P*. *villosa*. Solereder [15] also reported that "glandular shaggy hairs" have a multiseriate stipe. The stipe is mentioned to be variable in length, and when long, it has vascular bundles inside. The glandular "head" consists of a multi-seriated interior with elongated cells, which are the continuation of the stipe. A palisade secretory epidermis is also present, as verified for some species analyzed here classified into pattern II glands.

Killip [5] highlights that the sections *Dysosmia* (DC.) Killip and *Dysosmioides* Killip (subgenus *Passiflora*) have a petiole without a "true gland", although they often have gland-tipped hairs. On the other hand, in an extensive work on EFN in Passifloraceae, Cusset [34], named the structures found in *P*. *foetida* as glandular pseudo-hairs ("pseudo-poils glanduleux"). When assessing glandular development in this same species, Roth [20] concluded that they are EFN. Although the author did not mention any carbohydrate test, the presence of lipid droplets was observed in the secretory epidermis with a Sudan III test.

When analyzing *Passiflora foetida* cytologically and chemically, Durkee *et al*. [21] treated the glands of this species as resin glands. During the chemical tests, the authors did not find the presence of carbohydrates or amino acids as being secreted; however, they did react strongly with OsO4 *in situ*. The authors conclude that the glands present in *P*. *foetida* may represent a transition from true EFN to lipid-secreting glands, subsequently denominating them as resin glands.

The glands described in the present work as type II are small and elongated, with a cylindrical stipe, which can, after only a cursory analysis, be confused with trichomes, having an anatomical structure similar to that of glands described by Roth [20] and Durkee *et al*. [21]. This glandular type has been named by many authors as "glandular hairs" [2, 5, 8, 15, 34, 35]. However, Roth [20] has shown that it forms as subepidermal protuberances and thus called emergences by the author.

Apart from *Passiflora foetida* already described as having resin glands, *P*. *arida*, *P*. *sublanceolata* and *P*. *villosa* studied in the present work have secretory glands with very similar morphological and anatomical characteristics. Also, they did not show a positive reaction to the glucose strip test, suggesting that they are structurally similar to the resin gland in *Passiflora foetida*.

The section *Dysosmia* DC. is characterized by the presence of gland-tipped hairs, and *P*. *foetida* is coated by small sticky glands and sticky hairs [2, 8]. The absence of nectaries in *Dysosmia* species may be related to the presence of resin glands, a probable evolutionary novelty. However, this hypothesis needs to be verified in a phylogenetic context. A similar situation was reported by Conceição *et al*. [36], who verified that the EFN were replaced by sticky glandular hairs in a group within the *Chamaecrista* (Leguminosae). In the Bignonieae (Bignoniaceae) tribe, Nogueira *et al*. [37] verified that the evolution of glandular trichomes with viscous secretion contributes to the reduction of EFN, i.e., species with glandular trichomes have fewer EFN. The authors associate this substitution of EFN by glandular trichomes with the colonization of more arid environments, which would make the maintenance of EFN very costly for the plant, especially when considering the water availability.

In summary, this work reflects the initial stage of a much more extensive study of extrafloral secretory glands in *Passiflora*. The combined morphological and anatomical analyses, together with preliminary tests for the presence of glucose in the exudate of different *Passiflora* subgenera, suggests the occurrence of two categories of glands, resin glands and nectaries, the last one with a wide morphological diversity. The next challenges will involve determining the nectary histochemistry and ultrastructure, as well as the chemical nature of the exudate. Once the hypothesis of resin glands has been corroborated only in species of the *Dysosmia* section, we can think of a new interpretation of the evolution of the secretory glands in the group.

## Supporting information

S1 Fig Structural diversity of extrafloral nectaries (EFN) in *Passiflora* L. species, showing secretory region (arrow) in longitudinal section of glands. **A-C** Petiolar glands in P. *actinia*, *P*. *ambigua* and *P*. *coccinea*, respectively. **D** Gland of *P*. *coccinea* on abaxial surface of leaf blade. **E-F** Glands of *P*. *contracta* on petiole (E) and on abaxial surface of leaf blade (F). **G-H** Glands of *P*. *deidamioides* on petiole (G) and petiolule (H). **I-J** Glands of *P*. *edmundoi* on petiole (I) and at the margin of leaf blade (J). **K-L** Glands of *P*. *eichleriana* on petiole (K) and at the margin of leaf blade (L).
(TIF)

S2 Fig Continuation of S1 Fig. **A-B** Glands of *P*. *elegans* on petiole (A) and at the margin of leaf blade (B). **C-D**. Glands of *P*. *ferruginea* on petiole (C) and on abaxial surface of leaf blade (D). **E-F**. Glands of *P*. *galbana* on petiole (E) and at the margin of leaf blade (F). **G**. Gland of *P*. *gardneri* at the margin of leaf blade. **H-I**. Glands of *P*. *haematostigma* on petiole (H) and at the margin of leaf blade (I). **J-K**. Glands of *P*. *incarnata* on petiole (J) and at the margin of leaf blade (K). **L**. Gland of *P*. *kermesina* on petiole.
(TIF)

S3 Fig Continuation of S2 Fig. **A**. Gland of *P*. *kermesina* at the margin of leaf blade. **B-D**. Petiolar gland of *P*. *laurifolia*, *P*. *maliformis* and *P*. *ligularis*, respectively. **E-F** Glands of *P*. *miersii* at the margin of leaf blade (E) and petiole (F). **G**. Gland of *P*. *misera* on the abaxial surface of leaf blade. **H**. Petiolar gland of *P*. *morifolia*. **I-J** Glands of *P*. *odontophylla* on petiole (I) and at the margin of leaf blade (J).
(TIF)

S4 Fig Continuation of S3 Fig. **A**. Gland of *P*. *organensis* on abaxial surface of leaf blade. **B**. Petiolar gland of *P*. *racemosa*. **C-D**. Glands of *P*. *serratodigitata* on petiole (C) and at the margin of leaf blade (D). **E**. Petiolar gland of *P*. *setacea*. **F-G**. Glands of *P*. *sidifolia* on petiole (F) and at the margin of leaf blade (G). **H**. Petiolar gland of *P*. *suberosa*. **I-J**. Glands of *P*. *subrotunda* on petiole (I) and at the margin of leaf blade. **K** Gland of *P*. *umblicata* at the margin of leaf blade. **L**. Petiolar gland of *P*. *watsoniana*.
(TIF)

S1 Table Collection numbers of the analyzed species of *Passiflora* L. (DOCX)
