# Supplementary figures and images for: A structural review of foliar glands in Passiflora L. (Passifloraceae)
Source: PLoS One. 2017 Nov 14;12(11):e0187905. doi: 10.1371/journal.pone.0187905 (PMC5685584; doi:10.1371/journal.pone.0187905)

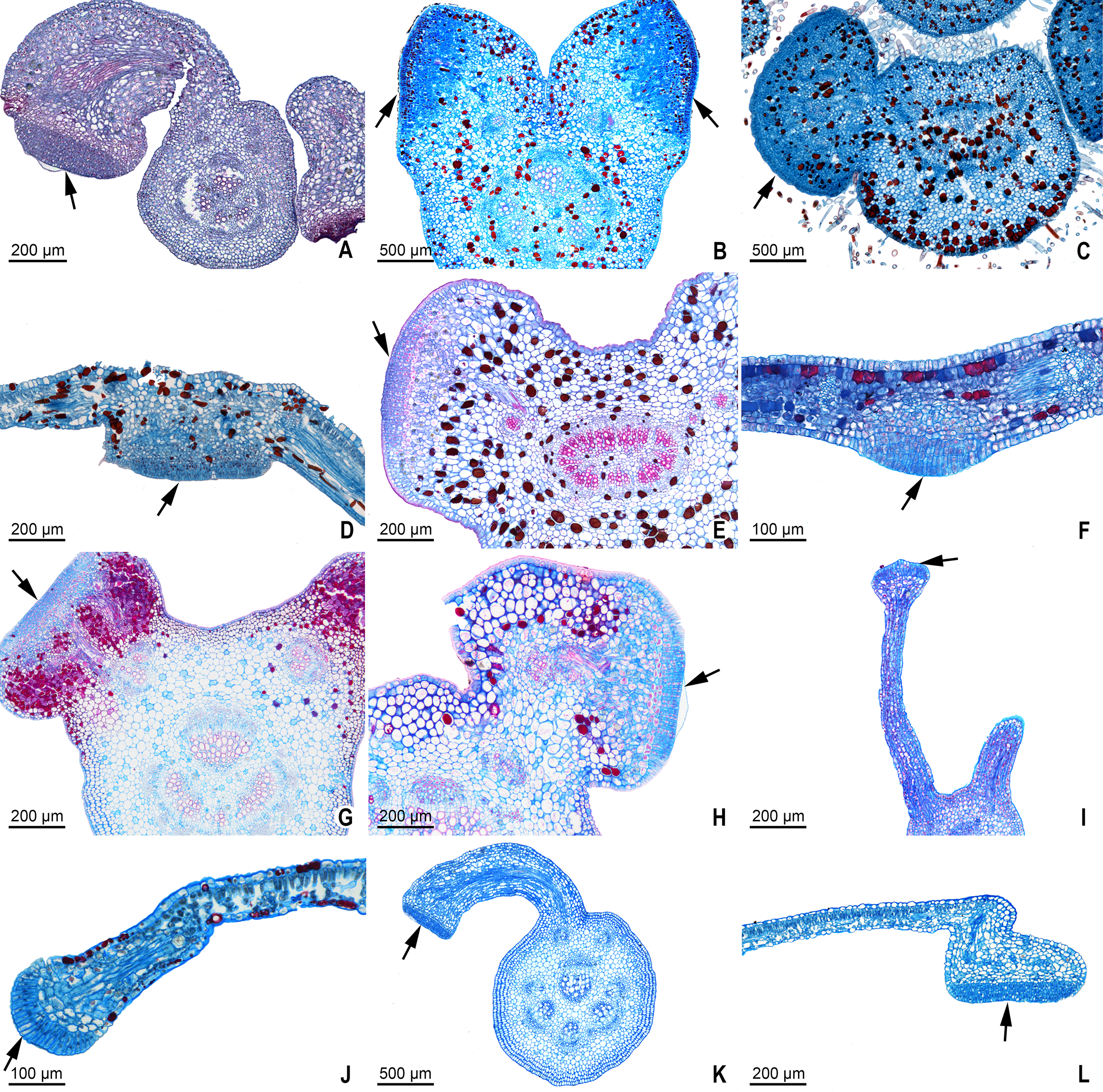

Supplement: S1 Fig — A-C Petiolar glands in P. actinia, P. ambigua and P. coccinea, respectively. D Gland of P. coccinea on abaxial surface of leaf blade. E-F Glands of P. contracta on petiole (E) and on abaxial surface of leaf blade (F). G-H Glands of P. deidamioides on petiole (G) and petiolule (H). I-J Glands of P. edmundoi on petiole (I) and at the margin of leaf blade (J). K-L Glands of P. eichleriana on petiole (K) and at the margin of leaf blade (L). (TIF) [file pone.0187905.s001.tif]

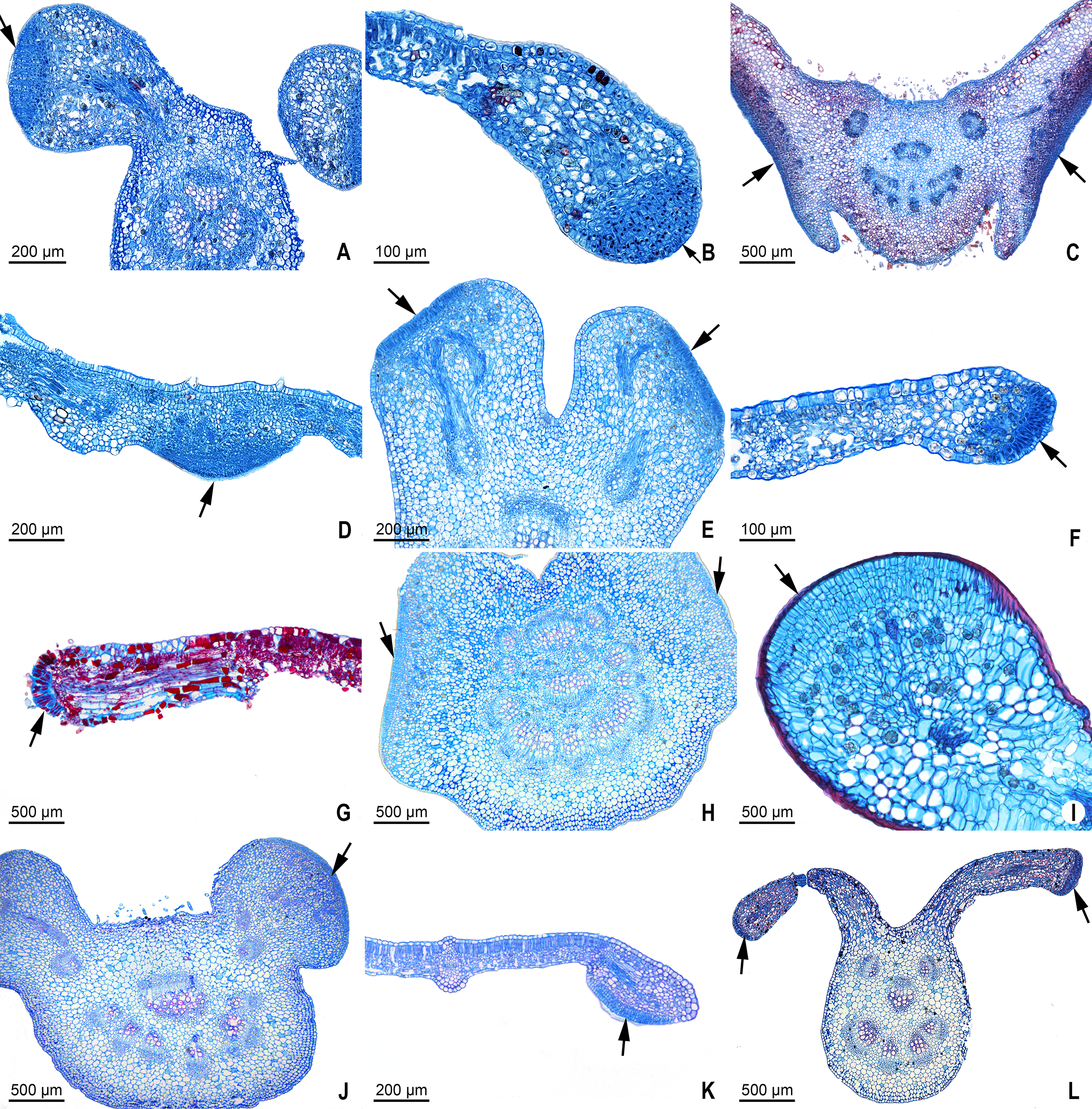

Supplement: S2 Fig — A-B Glands of P. elegans on petiole (A) and at the margin of leaf blade (B). C-D. Glands of P. ferruginea on petiole (C) and on abaxial surface of leaf blade (D). E-F. Glands of P. galbana on petiole (E) and at the margin of leaf blade (F). G. Gland of P. gardneri at the margin of leaf blade. H-I. Glands of P. haematostigma on petiole (H) and at the margin of leaf blade (I). J-K. Glands of P. incarnata on petiole (J) and at the margin of leaf blade (K). L. Gland of P. kermesina on petiole. (TIF) [file pone.0187905.s002.tif]

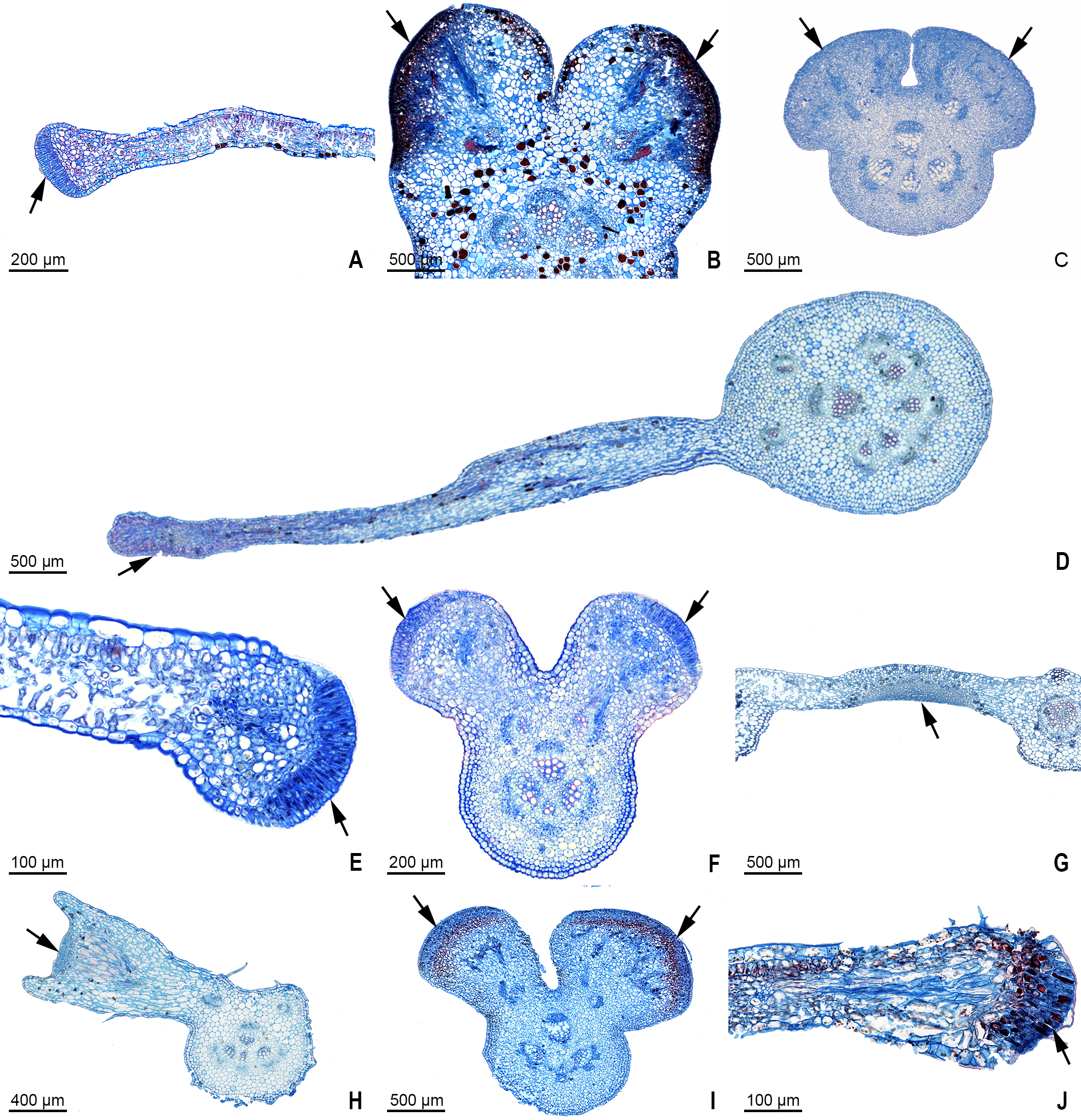

Supplement: S3 Fig — A. Gland of P. kermesina at the margin of leaf blade. B-D. Petiolar gland of P. laurifolia, P. maliformis and P. ligularis, respectively. E-F Glands of P. miersii at the margin of leaf blade (E) and petiole (F). G. Gland of P. misera on the abaxial surface of leaf blade. H. Petiolar gland of P. morifolia. I-J Glands of P. odontophylla on petiole (I) and at the margin of leaf blade (J). (TIF) [file pone.0187905.s003.tif]

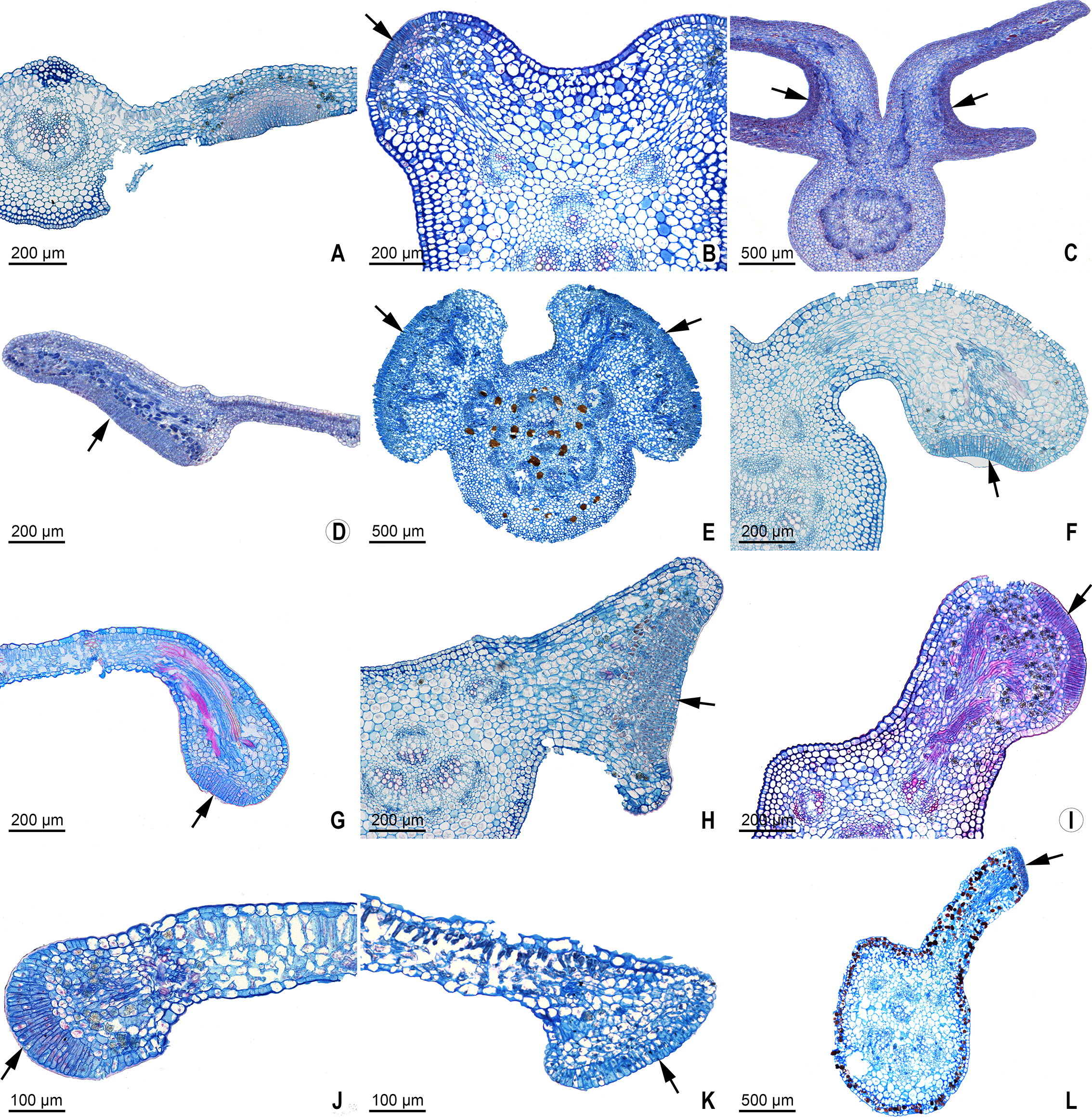

Supplement: S4 Fig — A. Gland of P. organensis on abaxial surface of leaf blade. B. Petiolar gland of P. racemosa. C-D. Glands of P. serratodigitata on petiole (C) and at the margin of leaf blade (D). E. Petiolar gland of P. setacea. F-G. Glands of P. sidifolia on petiole (F) and at the margin of leaf blade (G). H. Petiolar gland of P. suberosa. I-J. Glands of P. subrotunda on petiole (I) and at the margin of leaf blade. K Gland of P. umblicata at the margin of leaf blade. L. Petiolar gland of P. watsoniana. (TIF) [file pone.0187905.s004.tif]
