# Supplementary material for: A structural review of foliar glands in Passiflora L. (Passifloraceae)
Source: PLoS One. 2017 Nov 14;12(11):e0187905. doi: 10.1371/journal.pone.0187905 (PMC5685584; doi:10.1371/journal.pone.0187905)
Supplement: S1 Table — (DOCX) [file pone.0187905.s005.docx]

| **Species** | **Voucher**  **Voucher** |
| --- | --- |
| **Subgenus *Astrophea*** |  |
| *P. haematostigma* Mart. ex Mast. | R.C.C.Lemos 09 |
| **Subgenus *Decaloba*** |  |
| *P. ferruginea* Mast. | R.C.C.Lemos 43 |
| *P. misera* Kunth | R.C.C.Lemos 57 |
| *P. morifolia* Mast. | R.C.C.Lemos 62 |
| *P. organensis* Gardner | R.C.C.Lemos 51 |
| *P. suberosa* L. | R.C.C.Lemos 59 |
| **Subgenus *Deidamioides*** |  |
| *P. contracta* Vitta | R.C.C.Lemos 61 |
| *P. deidamioides* Harms | R.C.C.Lemos 31 |
| **Subgenus *Passiflora*** |  |
| *Passiflora actinia* Hook | R.C.C.Lemos 55 |
| *P. ambigua* Hemsl | M. Peixoto 65002 |
| *P. arida* (Mast. & Rose) Killip | R.C.C.Lemos 58 |
| *P. coccinea* Aubl. | R.C.C.Lemos 24 |
| *P. edmundoi* Sacco | R.C.C.Lemos 32 |
| *P. eichleriana* Mast. | R.C.C.Lemos 15 |
| *P. elegans* Mast. | R.C.C.Lemos 29 |
| *P. foetida* L. | R.C.C.Lemos 48 |
| *P. galbana* Mast. | R.C.C.Lemos 45 |
| *P. gardneri* Mast. | R.C.C.Lemos 44 |
| *P. incarnata* L. | R.C.C.Lemos 60 |
| *P. kermesina* Link & Otto | R.C.C.Lemos 50 |
| *P. laurifolia* L. | R.C.C.Lemos 18 |
| *P. ligularis* Juss. | R.C.C.Lemos 56 |
| *P. maliformis* L. | R.C.C.Lemos 30 |
| *P. miersii* Mart. | R.C.C.Lemos 37 |
| *P. odontophylla* Harms ex Glaz | R.C.C.Lemos 20 |
| *P. racemosa* Brot. | R.C.C.Lemos 4 |
| *P. serratodigitata* L. | R.C.C.Lemos 42 |
| *P. setacea* DC. | R.C.C.Lemos 26 |
| *P. sidifolia* M. Roem. | R.C.C.Lemos 17 |
| *P. sublanceolata* (Killip) MacDougal | R.C.C.Lemos 41 |
| *P. subrotunda* Mast. | R.C.C.Lemos 23 |
| *P. umbilicata* (Griseb.) Harms | R.C.C.Lemos 64 |
| *P. villosa* Vell. | R.C.C.Lemos 33 |
| *P. watsoniana* Mast. | R.C.C.Lemos 46 |
